# Effect of Capsaicin Stress on Aroma-Producing Properties of *Lactobacillus plantarum* CL-01 Based on E-Nose and GC–IMS

**DOI:** 10.3390/molecules29010107

**Published:** 2023-12-23

**Authors:** Qian Zhang, Junni Tang, Jing Deng, Zijian Cai, Xiaole Jiang, Chenglin Zhu

**Affiliations:** 1 College of Food Science and Technology, Southwest Minzu University, Chengdu 610041, China; zhangqian1121@outlook.com (Q.Z.); junneytang@swun.edu.cn (J.T.); caizijian@swun.edu.cn (Z.C.); 2 Cuisine Science Key Laboratory of Sichuan Province, Sichuan Tourism University, Chengdu 610100, China; dj3930590@sina.com; 3 College of Chemistry and Environment, Southwest Minzu University, Chengdu 610041, China; jiangxl@swun.edu.cn

**Keywords:** capsaicin stress, *Lactobacillus plantarum*, fermentation, volatile characteristics, chemometrics

## Abstract

Capsaicin stress, along with salt stress, could be considered the main stressors for lactic acid bacteria in traditional fermented pepper products. Until now, insufficient attention has been paid to salt stress, while the effect of capsaicin on the aroma-producing properties of *Lactobacillus plantarum* (*L. plantarum*) is unclear. The present study attempted to illustrate the effect of capsaicin stress on the aroma-producing properties of *L. plantarum* CL-01 isolated from traditionally fermented peppers based on E-nose and GC–IMS. The results showed that E-nose could clearly distinguish the overall flavor differences of *L. plantarum* CL-01 under capsaicin stress. A total of 48 volatile compounds (VOCs) were characterized by means of GC–IMS, and the main VOCs belonged to acids and alcohols. Capsaicin stress significantly promoted *L. plantarum* CL-01 to produce alpha-pinene, ethyl crotonate, isobutyric acid, *trans*-2-pentenal, 2-methyl-1-butanol, 3-methyl-3-buten-1-ol, 1-penten-3-one, 2-pentanone, 3-methyl-1-butanol-D, and 2-heptanone (*p* < 0.05). In addition, under capsaicin stress, the contents of 1-penten-3-one, 3-methyl-3-buten-1-ol, 5-methylfurfuryl alcohol, isobutanol, 2-furanmethanethiol, 2,2,4,6,6-pentamethylheptane, 1-propanethiol, diethyl malonate, acetic acid, beta-myrcene, 2-pentanone, ethyl acetate, *trans*-2-pentenal, 2-methylbutyl acetate, and 2-heptanone produced by *L. plantarum* CL-01 were significantly increased along with the fermentation time (*p* < 0.05). Furthermore, some significant correlations were observed between the response values of specific E-nose sensors and effective VOCs.

## 1. Introduction

Pepper, which belongs to the Capsicum genus, is considered one of the most necessary and economical vegetable crops and is widely cultivated around the world [1]. It is rich in various nutrients, such as vitamins, capsaicin, and minerals, and has antioxidant, anti-inflammatory, and anti-obesity properties [2]. Therefore, pepper is not only consumed as food to provide a spicy taste but is also used as an effective component to produce medicines [3]. In China, several traditional fermented peppers with local characteristics were produced due to the complex ecological environment and climatic conditions in different areas [4]. For instance, Doubanjiang [5], Chili paste [6], and *Paojiao* [7] are favored by consumers due to their distinct flavor and mild pungency [8].

Volatile compounds (VOCs) are crucial to provide flavor to fermented products, which are mainly derived from microbial metabolism during fermentation [9]. As one of the main microorganisms in fermented peppers, lactic acid bacteria (LAB) have the ability to produce various VOCs through carbohydrate metabolism, amino acid catabolism, and fatty acid metabolism and, in turn, leads to the unique flavor of various fermented peppers [10]. López-Salas et al. found that consumers preferred Habanero pepper fermented with commercial or wild *Lactobacillus plantarum* (*L. plantarum*) compared to unfermented ones, and the fermented product showed significantly higher levels of VOCs, such as 1-hexanol, *cis*-3-hexenyl hexanoate, and 3,3-dimethylhexan-1-ol [11]. Moreover, a previous study suggested that *Lactobacillus*, the dominant microbiota in salted fermented peppers, was positively correlated with the contents of valencene, hexyl acetate, and toluene, and these compounds conferred fruity, floral, and woody aromas to products [12]. Therefore, the aroma-producing ability of LAB is essential to attribute the flavor characterizations of the final pepper products. However, LAB is exposed to many adverse conditions during pepper fermentation, and its metabolic pathways undergo a series of changes [13]. Currently, there are a few studies on the effects of stress conditions, such as acid, salt, and thermal conditions, on the alterations of LAB metabolism [14]. Capsaicin, derived from pepper, could affect the metabolomic feature of LAB, which could be granted by its antibacterial properties. Therefore, the quality of fermented pepper products can change due to distinct LAB activities under capsaicin stress [6].

Gas chromatography–ion mobility spectrometry (GC–IMS) combines the high separation capability of GC and the fast response of IMS, which solves the drawbacks that exist in GC-MS and provides an effective method for the separation and sensitive detection of VOCs [15,16]. Gallegos et al. found that GC–IMS could rapidly characterize VOCs from LAB and that the physiological state of strains could be reflected by the IMS signal intensity during cheese fermentation [15]. Electronic nose (E-nose), an artificial sensory technology that simulates the human smell mechanism, is equipped with a range of chemical sensors to effectively distinguish VOCs [17,18]. Arnold and Senter found that the different bacterial species had different VOC areas by means of the E-nose [19]. In addition, the combination of E-nose and GC–IMS was reported to provide a comprehensive and accurate view of characterizing the VOCs in foods and thereby could assess their flavor profiles [16]. However, rare attention has been paid to exploring the changes in VOCs of LAB under capsaicin stress by combining E-nose and GC–IMS.

In order to fill such a gap, capsaicin, instead of pepper directly, was used to explore its effect on aroma-producing property changes in LAB so as to remove the disturbances of other endogenous substances in the pepper. Moreover, *L. plantarum* CL-01, isolated from traditionally fermented peppers, was involved in analyzing its alterations of aroma-producing properties under capsaicin stress by combining E-nose and GC–IMS. Moreover, the correlations between the concentrations of VOCs and response values of E-nose sensors were set. The present results could provide reference data for the aroma-producing properties of *L. plantarum* under capsaicin stress and shed light on the LAB strain screening for the quality promotion of fermented pepper products.

## 2. Results

### 2.1. Effect of Different Capsaicin Concentrations on the Aroma-Producing Properties of L. plantarum

#### 2.1.1. E-Nose Analysis

**Figure 1:**
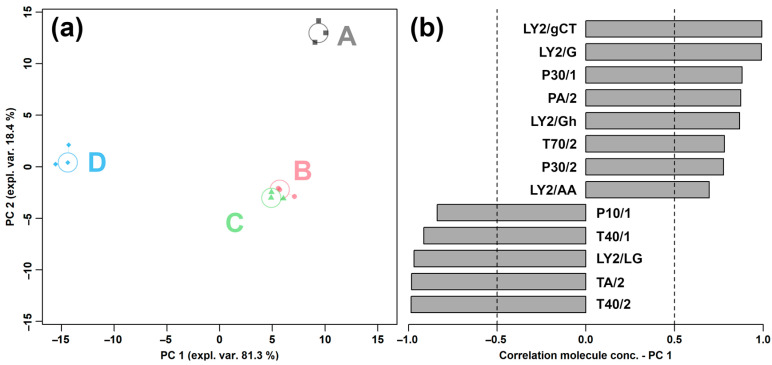
rPCA model for E-nose analysis of *L. plantarum* under different capsaicin concentrations, representing Scoreplot (**a**) and Loading plot (**b**). A, B, C, and D represent the four groups, whose capsaicin concentrations are 0, 0.125, 0.25, and 0.5 mg/mL, respectively.

The results of the response values of E-nose sensors to *L. plantarum* under different capsaicin concentrations are displayed in Table S1. To obtain the overall flavor profile, the response values of all E-nose sensors were used as the basis for a robust principal component analysis (rPCA) model. The result is shown in Figure 1.

In Figure 1a, PC 1 accounted for 81.3% of the variance of the entire samples’ set, thus nicely summarizing the overall flavor characteristics of *L. plantarum* under different capsaicin concentrations. In detail, group A showed the highest response values of LY2/gCT, LY2/G, P30/1, PA/2, LY2/Gh, T70/2, P30/2, and LY2/AA, and the lowest response values of P10/1, T40/1, LY2/LG, TA/2, and T40/2 among the four groups.

#### 2.1.2. GC–IMS Analysis

**Figure 2:**
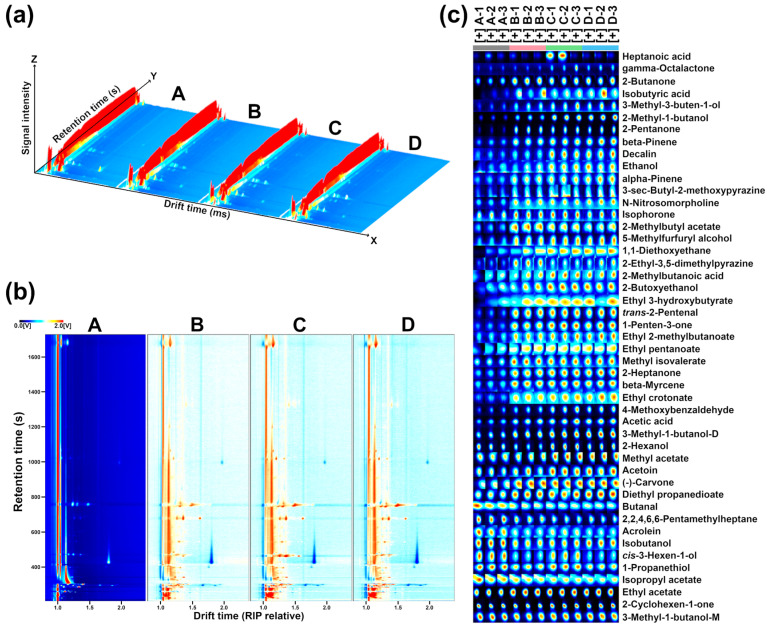
GC–IMS observation of *L. plantarum* under different capsaicin concentrations. Three-dimensional topographic plot (**a**); 2D difference plot (**b**); Gallery plots indicating the variations of VOCs’ relative content among the four groups (**c**). Red and blue colors underline over- and under-expressed components in both (**b**,**c**).

Figure 2 summarizes the processing pipeline of GC–IMS information on the VOCs produced by *L. plantarum* under different capsaicin concentrations.

The 3D topographic graph provides a visual representation of how the flavor profiles of *L. plantarum* differ in various parts of the GC–IMS spectrum under different capsaicin concentrations. The 2D difference plot shows the point-by-point differences among groups. The gallery plot shows that the distinct flavor profile of VOCs mainly pertains to acids and alcohols among the groups. A total of 48 VOCs were characterized in all samples, which were divided into six categories, namely, aldehydes (4), acids (4), ketones (8), esters (11), alcohols (12), and others (9). 

**Figure 3:**
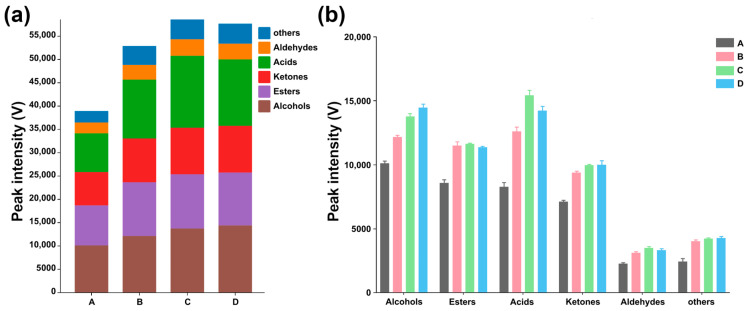
Peak intensities of each category of VOCs produced by *L. plantarum* fermented for 24 h under different capsaicin concentrations. (**a**) Peak intensity of total VOCs among the four groups. (**b**) Peak intensities of each category of VOCs among the four groups.

Figure 3 illustrates the changes in peak intensities of VOCs produced by *L. plantarum* after 24 h fermentation under different capsaicin concentrations. The relevant information on VOCs of *L. plantarum* fermented for 24 h under different capsaicin concentrations is presented in Table 1.

**Figure 4:**
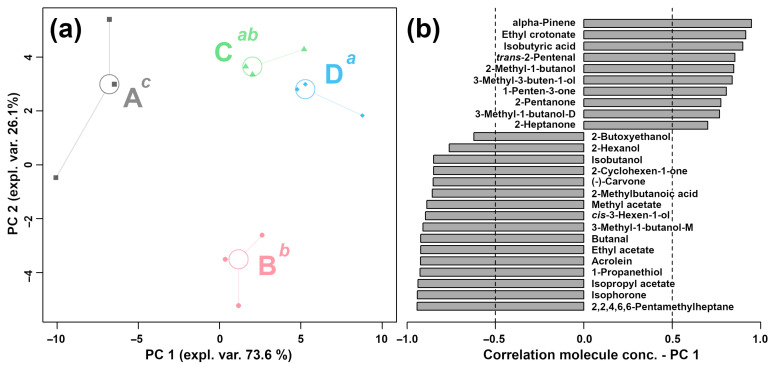
The rPCA model calculated on the basis of VOCs shows significant differences in peak intensities among *L. plantarum* samples under different capsaicin concentrations. Scoreplot (**a**) displays the overall structure of the data. A, B, C, and D represent the four groups, whose capsaicin concentrations are 0, 0.125, 0.25, and 0.5 mg/mL, respectively. Superscript lowercase letters indicate the significance of samples along PC 1. Loading plot (**b**) displays significant correlation between the peak intensity of each VOC and the importance over PC 1 (*p* < 0.05).

Among the characterized VOCs, 38 VOCs showed significant differences among the four groups. An rPCA model was built according to the peak intensities of VOCs to highlight the overall trend of the flavor profile (Figure 4).

Compared to A, the B, C, and D groups were found to be mainly characterized by higher contents of alpha-pinene, ethyl crotonate, isobutyric acid, *trans*-2-pentenal, 2-methyl-1-butanol, 3-methyl-3-buten-1-ol, 1-penten-3-one, 2-pentanone, 3-methyl-1-butanol-D, and 2-heptanone and lower contents of 2-butoxyethanol, 2-hexanol, isobutanol, 2-cyclohexen-1-one, (-)-carvone, 2-methylbutanoic acid, methyl acetate, *cis*-3-hexen-1-ol, 3-methyl-1-butanol-M, butanal, ethyl acetate, acrolein, 1-propanethiol, isopropyl acetate, isophorone, and 2,2,4,6,6-pentamethylheptane.

**Figure 5:**
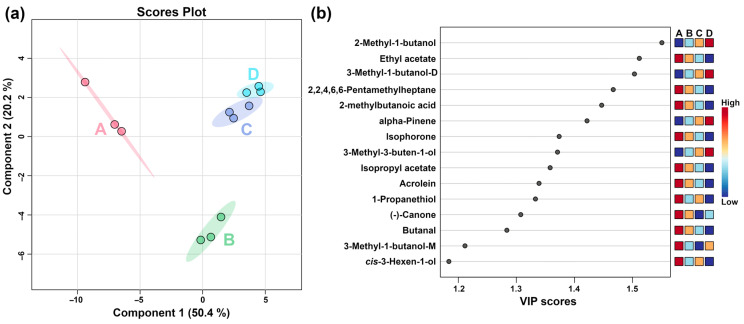
PLS-DA of VOCs produced by *L. plantarum* samples under different capsaicin concentrations. (**a**) Score plot. (**b**) VIP scores. A, B, C, and D represent capsaicin concentrations of 0, 0.125, 0.25, and 0.5 mg/mL, respectively.

To further obtain the key VOCs produced by *L. plantarum* fermentation under capsaicin stress, PLS-DA was performed on the VOCs, and the result is shown in Figure 5.

In Figure 5a, component one accounted for 50.4% of the total variance and is well-discriminated. Figure 5b shows VIP scores of the top 15 VOCs, namely, 2-methyl-1-butanol, ethyl acetate, 3-methyl-1-butanol-D, 2,2,4,6,6-pentamethylheptane, 2-methylbutanoic acid, alpha-pinene, isophorone, 3-methyl-3-buten-1-ol, isopropyl acetate, acrolein, 1-propanethiol, (-)-Canone, butanal, 3-methyl-1-butanol-M, and *cis*-3-hexen-1-ol.

#### 2.1.3. Correlation between E-Nose and GC–IMS

**Figure 6:**
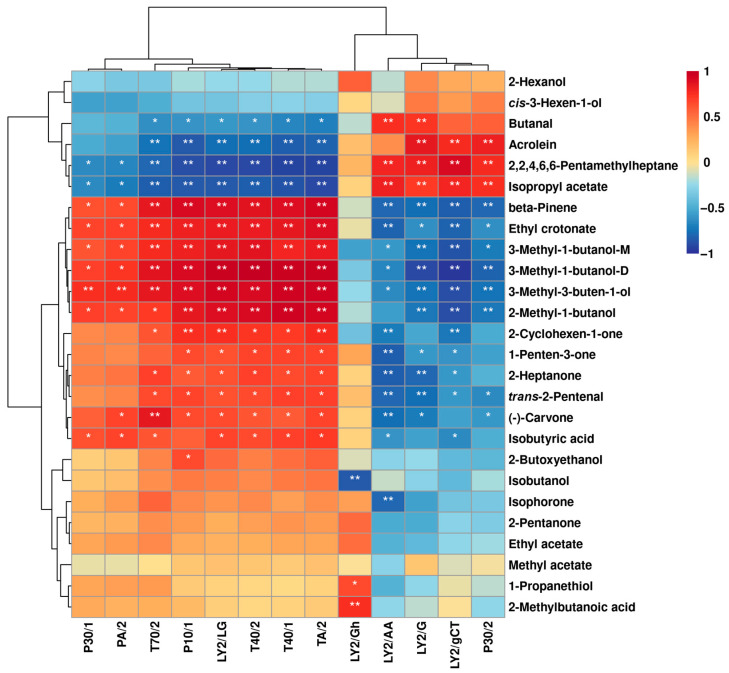
Spearman’s correlation heatmap displays the correlation between E-nose sensor responses and VOCs’ contents of *L. plantarum* under different capsaicin concentrations. Each color represents the correlation coefficient, with blue and red indicating negative and positive correlations. “*” and “**” represent significance at *p* < 0.05 and *p* < 0.01, respectively.

The aroma-producing properties of *L. plantarum* under different capsaicin concentrations were analyzed from distinct perspectives by means of E-nose and GC–IMS. E-nose provided the overall information on VOCs among the groups, while GC–IMS provided the detailed profile of VOCs. The correlation between E-nose sensor response values and peak intensities of VOCs detected by GC–IMS is shown in Figure 6.

The LY2/LG, T40/2, T40/1, and TA/2 sensors were positively associated with ethyl crotonate, isobutyric acid, *trans*-2-pentenal, 2-methyl-1-butanol, 3-methyl-3-buten-1-ol, 1-penten-3-one, 3-methyl-1-butanol-D, and 2-heptanone. These VOCs were found at higher levels in A compared to the other three groups. The P10/1, LY2/LG, T40/2, T40/1, and TA/2 sensors were positively associated with 2-cyclohexen-1-one, (-)-carvone, and 3-methyl-1-butanol-M but negatively associated with butanal, acrolein, isopropyl acetate, and 2,2,4,6,6-pentamethylheptane. Compared to the other three groups, D contained higher levels of these VOCs.

### 2.2. Effect of Capsaicin Stress on the Aroma-Producing Properties of L. plantarum during Fermentation

#### 2.2.1. E-Nose Analysis

**Figure 7:**
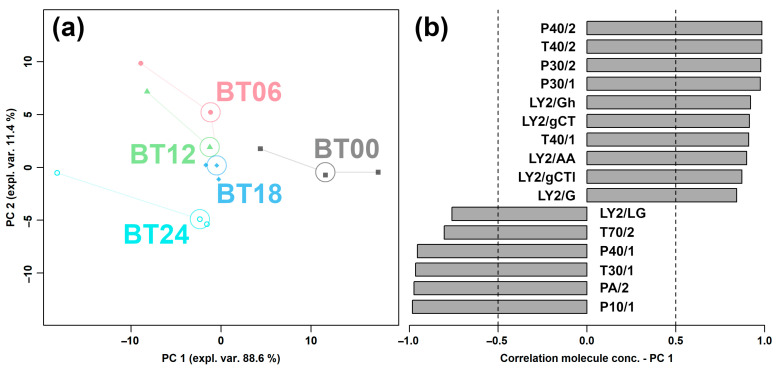
rPCA model for E-nose analysis of *L. plantarum* along fermentation under capsaicin stress, representing Scoreplot (**a**) and Loading plot (**b**).

Table S2 presents the results of the response values of the E-nose sensor to *L. plantarum* under capsaicin stress at different fermentation times. Similar to the above issues, to obtain the effect of capsaicin stress on the overall flavor of *L. plantarum* during fermentation, the response values of all E-nose sensors were used as the basis for an rPCA model, as shown in Figure 7.

In Figure 7a, PC 1 accounted for 88.6% of the variance of the entire samples’ set, thus nicely summarizing the overall characteristics of the *L. plantarum* samples during fermentation under capsaicin stress. In detail, BT00 showed significantly higher response values of P40/2, T40/2, P30/2, P30/1, LY2/Gh, LY2/gCT, T40/1, LY2/AA, LY2/gCTI, and LY2/G and significantly lower response values of LY2/LG, T70/2, P40/1, T30/1, PA/2, and P10/1.

#### 2.2.2. GC–IMS Analysis

**Figure 8:**
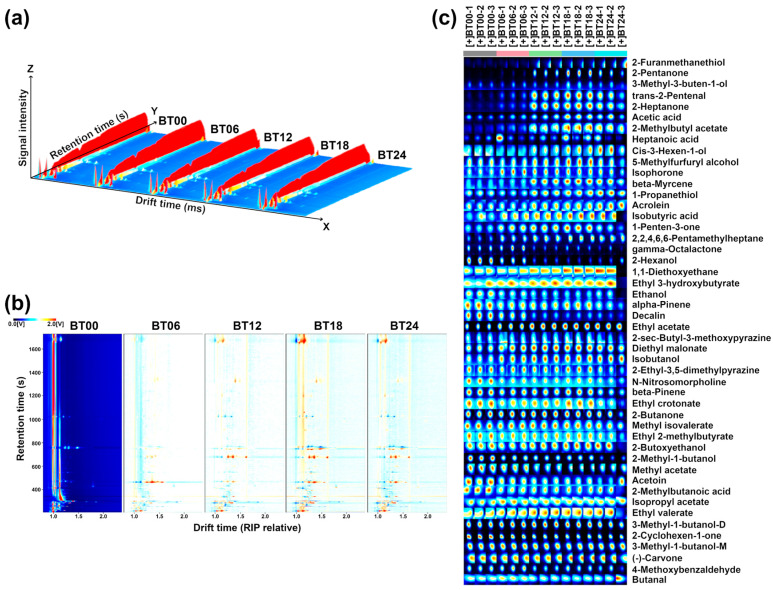
GC–IMS observation of *L. plantarum* along fermentation under capsaicin stress. Three-dimensional topographic plot (**a**); 2D difference plot (**b**); Gallery plots indicating the variations of VOCs’ relative content among the five groups (**c**). Red and blue colors underline over- and under-expressed components in both (**b**,**c**).

Figure 8 summarizes the processing pipeline of GC–IMS information on the VOCs produced by *L. plantarum* during fermentation under capsaicin stress.

**Figure 9:**
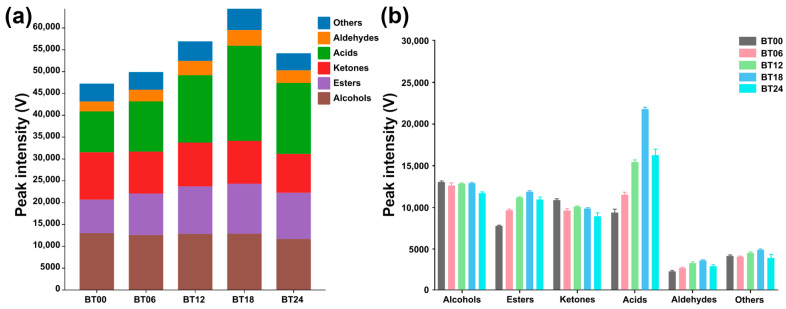
Peak intensities of each category of VOCs produced by *L. plantarum* fermented at different times under a certain capsaicin concentration. (**a**) Peak intensity of total VOCs among the five groups. (**b**) Peak intensities of each category of VOCs among the five groups.

The gallery plot (Figure 8c) showed that VOCs of *L. plantarum* during fermentation under capsaicin stress mainly pertained to acids and alcohol. Compared to BT00, the total relative content of VOCs was increased in BT06, BT12, BT18, and BT24, as shown in Figure 9. The peak intensities of VOCs among the groups are shown in Table 2.

**Figure 10:**
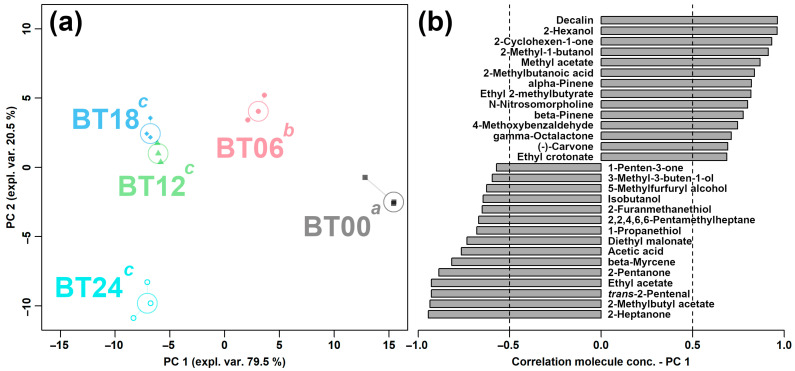
The rPCA model calculated on the basis of VOCs shows significant differences in peak intensities among groups. Scoreplot (**a**) displays the overall structure of the data. Superscript lowercase letters indicate the significance of samples along PC 1. Loading plot (**b**) displays significant correlation between the peak intensity of each VOC and the importance over PC 1 (*p* < 0.05).

Among the above-characterized VOCs, 35 VOCs showed significant differences among the five groups. Similar to the above condition, an rPCA model was built, as shown in Figure 10.

Compared to BT00, BT06, BT12, BT18, and BT24 were found to be mainly characterized by higher contents of 1-penten-3-one, 3-methyl-3-buten-1-ol, 5-methylfurfuryl alcohol, isobutanol, 2-furanmethanethiol, 2,2,4,6,6-pentamethylheptane, 1-propanethiol, diethyl malonate, acetic acid, beta-myrcene, 2-pentanone, ethyl acetate, *trans*-2-pentenal, 2-methylbutyl acetate, and 2-heptanone and by lower contents of decalin, 2-hexanol, 2-cyclohexen-1-one, 2-methyl-1-butanol, methyl acetate, 2-methylbutanoic acid, alpha-pinene, ethyl 2-methylbutyrate, N-nitrosomorpholine, beta-pinene, 4-methoxybenzaldehyde, gamma-octalactone, (-)-carvone, and ethyl crotonate.

**Figure 11:**
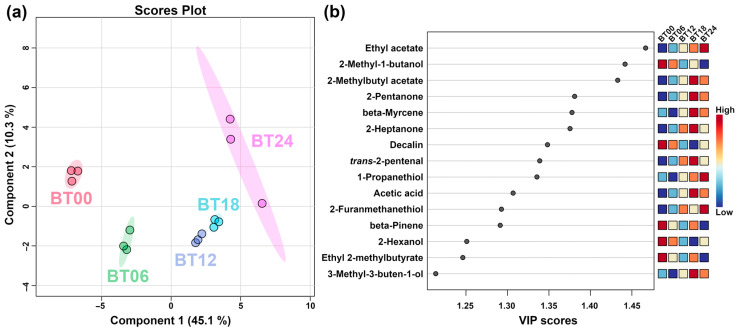
PLS-DA of VOCs produced by *L. plantarum* samples fermentation at different fermentation times under capsaicin stress. (**a**) Score plot. (**b**) VIP scores.

To further obtain the key VOCs produced by *L. plantarum* fermentation at different fermentation times under capsaicin stress, PLS-DA was performed on the VOCs, and the result is shown in Figure 11.

In Figure 11a, component one accounted for 45.1% of the total variance and is well-discriminated. Figure 11b shows VIP scores of the top 15 VOCs, namely, ethyl acetate, 2-methyl-1-butanol, 2-methylbutyl acetate, 2-pentanone, beta-myrcene, 2-heptanone, decalin, *trans*-2-pentenal, 1-propanethiol, acetic acid, 2-furanmethanethiol, beta-pinene, 2-hexanol, ethyl 2-methylbutyrate, and 3-methyl-3-buten-1-ol.

#### 2.2.3. Correlation between E-Nose and GC–IMS

**Figure 12:**
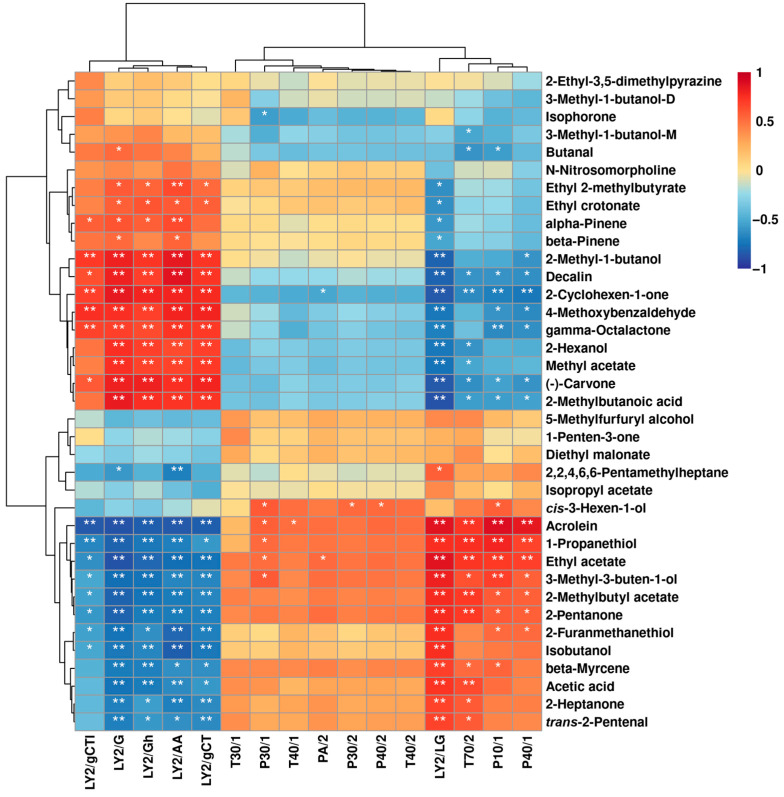
Spearman’s correlation heatmap displays the correlation between E-nose sensor responses and VOCs’ contents. Each color represents the correlation coefficient, with blue and red indicating negative and positive correlations. “*” and “**” represent significance at *p* < 0.05 and *p* < 0.01, respectively.

Figure 12 presents the correlation between the E-nose sensor response values and peak intensities of VOCs detected by GC–IMS of *L. plantarum* during fermentation under capsaicin stress.

The LY2/G, LY2/Gh, LY2/AA and LY2/gCT sensors were positively associated with *beta*-pinene, 2-methyl-1-butanol, decalin, 2-cyclohexen-1-one, 4-methoxybenzaldehyde, gamma-octalactone, 2-hexanol, methyl acetate, (-)-carvone, and 2-methylbutanoic acid but negatively associated with acrolein, 1-propanethiol, ethyl acetate, 3-methyl-3-buten-1-ol, 2-methylbutyl acetate, 2-pentanone, 2-furanmethanethiol, isobutanol, beta-myrcene, acetic acid, 2-heptanone, and *trans*-2-pentenal. LY2/LG, T70/2, P10/1, and P40/1 showed positive connections to acrolein, 1-propanethiol, ethyl acetate, 3-methyl-3-buten-1-ol, 2-methylbutyl acetate, and 2-pentanone but negative connections to decalin, 2-cyclohexen-1-one, (-)-carvone, and 2-methylbutanoic acid.

## 3. Discussion

Flavor is a vital indicator of the quality of fermented products and the choice of purchase by consumers [20]. The flavor of fermented peppers is mainly due to the dominant microorganisms, such as LAB, that can produce various VOCs through complex biochemical reactions. However, the effect of capsaicin stress on the aroma-producing properties of *L. plantarum* is unclear. Therefore, this study attempted to investigate the effect of capsaicin stress on the flavor profile of *L. plantarum* by means of E-nose and GC–IMS.

The response of the E-nose sensors showed significant differences for *L. plantarum* with different capsaicin concentrations and fermentation times, and combined with the rPCA model, they were clearly distinguishable among the samples. This could indicate that E-nose is highly sensitive to the flavor of samples and clearly reflects the differences in the overall flavor profile of *L. plantarum* fermented under capsaicin stress. However, E-nose fails to identify specific VOCs that contribute to the overall response. Therefore, high-throughput techniques, such as GC–IMS, should be combined to capture fine-grained information [21].

Acids are primarily produced by LAB during fermentation, which could serve as contributors to the flavor of fermentation products. In our study, acids were the most abundant VOCs characterized by GC–IMS. In addition, capsaicin stress could promote *L. plantarum* to produce acids. Such a result might be attributed to the alternation of metabolism of *L. plantarum* under capsaicin stress, which, in turn, could change the flavor profile. The generation of acetic acid is achieved through the metabolism of citric acid and the acetate kinase pathway [22]. Acetic acid plays a key role in fermented foods and is attributed to the main characteristic flavors, such as a pungent, sharp, and vinegary flavor [23]. Gao et al. compared the effects of LAB fermentation on a seaweed sauce flavor by means of GC–IMS. The results showed that LAB fermentation led to a higher content of acetic acid, which provided the seaweed sauce with a more refreshing and distinctive flavor profile [24]. 

Alcohols, as essential metabolites of LAB during fermentation, are generated primarily through redox reactions of unsaturated ketones or aldehydes and amino acid metabolic pathways [25]. As precursors of many biochemical reactions in fermentation products, alcohols not only have a pleasant fruity, floral, and malty aroma but also serve as solvents for other VOCs and, therefore, play important roles in the flavor formation of products [26]. In the present study, capsaicin stress promoted *L. plantarum* to produce higher levels of 2-methyl-1-butanol and 3-methyl-3-buten-1-ol, and both of these were key VOCs produced by *L. plantarum* fermentation under capsaicin stress. 2-Methyl-1-butanol is the organic compound derived from isoleucine and its corresponding aldehyde [27], which has a mildly aromatic and pungent flavor and was found to be a key flavor volatile in Doubanjiang [26]. 3-Methyl-3-buten-1-ol is the enol organic compound produced by LAB metabolism, with a sweet and fruity flavor [28]. The above results suggested that capsaicin stress could alter the metabolism of *L. plantarum* and, in turn, regulate VOC production. Meanwhile, the levels of isobutanol, 5-methylfurfuryl alcohol, 2-furanmethanethiol, and 1-propanethiol increased during fermentation, and all of these VOCs could confer good flavor to the fermented products. In particular, isobutanol not only could react with acids to form esters but also provided a harmonious mouthfeel and distinctive full-bodied flavor to the fermented product. 

Esters are mainly formed by the esterification of acids and alcohols. They are essential contributors to the flavor of fermented peppers due to their low threshold and fruity and floral aroma [29]. In our study, the major esters produced by *L. plantarum* under capsaicin stress were ethyl acetate, ethyl crotonate, diethyl malonate, and 2-methylbutyl acetate. In particular, ethyl acetate, which provides a sweet and fruity flavor, is the key VOC in fermented peppers. Wang et al. found that inoculation of *Lactobacillus* isolated from naturally fermented chopped peppers could increase the content of ethyl acetate in fermented peppers with a better flavor [30].

Similar to esters, aldehydes and ketones also have a low odor threshold and contribute significantly to the overall flavor of the fermentation product [31]. They are mainly derived from fatty acid oxidation and the Strecker degradation pathway of amino acids [32]. Moreover, such compounds are the main sources of flavor and essential VOCs in fermented peppers [33]. In general, aldehydes possess pleasant, malty, fruity, and grassy flavors [9], and ketones facilitate the fruit, mushroom, and wood flavors [34]. It was reported that the metabolic activity of LAB during fermentation may be altered by the high content of aldehydes and ketones in *Paojiao* [7]. Combined with our results, *L. plantarum* fermented under capsaicin stress produced higher levels of aldehydes and ketones, which may indicate that capsaicin stress could promote the metabolism of *L. plantarum*. Notably, the content of *trans*-2-pentenal generated by *L. plantarum* increased significantly with an increasing capsaicin concentration and fermentation time, which gave the typical fruity flavor to fermented products. Moreover, the levels of 1-penten-3-one, 2-pentanone, and 2-heptanone were significantly increased under capsaicin stress. 1-Penten-3-one, a strong pungent odor indicator, is the main VOC for the characteristic odor of peppers [35]. With the help of GC–IMS, we detected a higher level of 1-penten-3-one in *L. plantarum* samples under capsaicin stress. 2-Pentanone is described as fruity, sweet, and banana-flavored [35]. Li et al. investigated the flavor profile of *L. plantarum* PC8 fermented, dried, and fresh chili sauces by HS-SPME-GC-MS and found that the fermented and dried chili exhibited a unique nutty, herbal, and fermented flavor profile and identified 2-heptanone as the characteristic flavor component [25]. Therefore, the increased content of 2-pentanone and 2-heptanone in *L. plantarum* under capsaicin stress suggested that capsaicin stress could help to promote the aroma production ability of *L. plantarum*, which, in turn, enriched the flavor of the fermented samples.

## 4. Materials and Methods

### 4.1. Activation of Bacteria

*L. plantarum* CL-01 was isolated from traditional fermented Ciba pepper, identified by 16S rDNA sequencing, and stored at the Microbiology Laboratory of the College of Food Science and Technology, Southwest Minzu University. The strain was activated in De Man, Rogosa, and Sharpe (MRS) broth at 37 °C for 24 h and cultured with 2% (*v*/*v*) inoculum for 24 h. Finally, a suspension of *L. plantarum* CL-01 was obtained with concentration of 10^9^ CFU/mL.

### 4.2. Preparation of Capsaicin Solution

Standard solutions of capsaicin were prepared according to the method of Sabela et al. [36]. In brief, the standard solution of 0.5 mg/mL was prepared by dissolving 50 mg of capsaicin standard powder in 100 mL of absolute ethanol (99.9% purity). Further diluted with absolute ethanol to make capsaicin solutions at concentrations of 0.25 and 0.125 mg/mL. All capsaicin solutions were stored at 4 °C.

### 4.3. Fermentation

**Figure 13:**
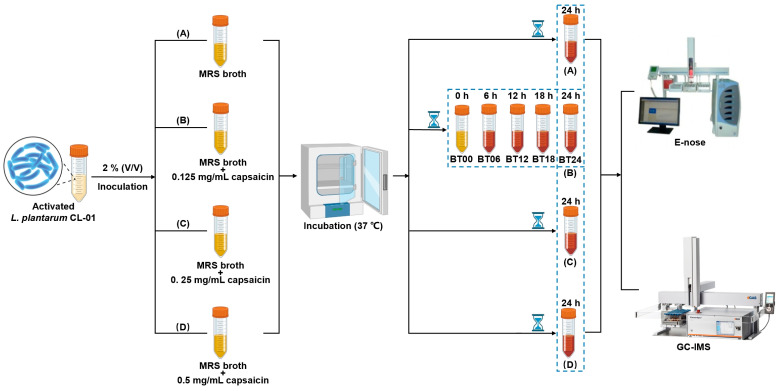
A scheme of experimental design.

The activated *L. plantarum* CL-01 were inoculated into MRS broth with concentrations of 0 (A), 0.125 (B), 0.25 (C), and 0.5 (D) mg/mL capsaicin at an inoculum of 2% (*v*/*v*), respectively, and left to incubate at 37 °C for 24 h. In addition, activated *L. plantarum* CL-01 was inoculated into MRS broth with 0.125 mg/mL capsaicin at an inoculum of 2% (*v*/*v*) and incubated at 37 °C for 0 h (BT00), 6 h (BT06), 12 h (BT12), 18 h (BT18), and 24 h (BT24), as shown in Figure 13.

### 4.4. E-Nose Analysis

According to the method of Zhao et al. with appropriate optimization [21]. An E-nose system (FOX 4000, Alpha MOS, Toulouse, France) was used to detect VOCs in *L. plantarum* samples. Following Wen et al. [37], the system contains 18 metal oxide sensors, and the main information provided by each sensor is shown in Table 3. To meet the requirements of the E-nose analysis, 2 mL of sample was placed in a 10 mL headspace vial, and then the sample was incubated at 75 °C for 5 min and manually injected with a 500 μL injection volume. The test conditions for E-nose analysis were set as follows: the flow rate was 150 mL/s for the carrier gas (synthetic dry air); the measurement time for each sample was 120 s; the recovery time was 300 s. To gain stable data, measurements were repeated five times for each sample, and the last three sets of data were retained. The average of the three stable sets was used for the following analysis.

### 4.5. GC–IMS Analysis

According to the method of Zhang et al. with appropriate optimization [38]. The VOCs of *L. plantarum* culture samples were characterized by a GC–IMS (Flavorspec^®^, G.A.S. Instrument, Munich, Germany) with an MXT-WAX capillary column (30 m × 0.53 mm × 1 μm) (Restek, Mount Ayr, IN, USA). Each sample (1.5 mL) was taken to a 20 mL headspace vial with a magnetic screw seal cover and incubated for 10 min at 50 °C. Then, headspace sample (100 μL) was automatically injected into the injector (no shunt mode) with the help of heated syringe at 85 °C. The temperatures of column and drift tube were maintained at 60 °C and 45 °C, respectively. A total of 150 mL/min was applied for drift gas flow rate. A high-purity N_2_ (99.999% purity) was used. The GC column flow rate was programmed in line with the following program: 2 mL/min for 5 min, 10 mL/min for 10 min, 15 mL/min for 5 min, 50 mL/min for 10 min, and 100 mL/min for 10 min. As suggested by previous studies [39,40], the retention index (RI) of VOCs was calculated by means of n-ketone C4–C9 as a reference. In order to identify detected VOCs, their RI and ions’ drift times were compared with those of the standards in the GC–IMS library. Each sample was tested once. The relative quantification of each VOC was based on its peak intensity. The plots, namely three-dimensional (3D) topographic plots, two-dimensional (2D) difference plots, and gallery plots, were built taking advantage of the Laboratory Analytical Viewer, Reporter, and Gallery Plot supplemented with GC–IMS instrument.

### 4.6. Statistical Analysis

Statistical analysis was performed using the R computational language. Prior to univariate analysis, the data obtained by E-nose and GC–IMS were pre-processed in two steps. First, the data were normalized by means of probabilistic quotient normalization (PQN) so as to remove the effects of several confounding factors, such as extremely high contents of molecules or response values of sensors. Second, the nonnormally distributed data were transformed into normally distributed according to Box and Cox [41]. In order to find out the significant differences among groups, ANOVA, followed by Tukey HSD test, was applied by taking advantage of “aov” function of the “stats” of R package. For this purpose, a cut-off *p*-value of 0.05 was accepted.

To obtain the overall trend of the flavor profile of the samples, all sensors of E-nose and the VOCs with significant differences were used as basis for building the rPCA model by means of “agricolae” package. For each rPCA model, we calculated the Scoreplot and Pearson correlation plot based on the loadings. In addition, MetaboAnalyst 5.0 was used for PLS-DA of VOCs characterized by GC–IMS. Spearman correlation analysis between the E-nose and GC–IMS was setup by means of an online tool (https://www.omicstudio.cn, accessed on 25 October 2023).

## 5. Conclusions

To the best of our knowledge, the present study, for the first time, attempted to investigate the effect of capsaicin stress on the aroma-producing properties of *L. plantarum* through the combination of E-nose and GC–IMS. Both techniques could be considered rapid methods for identifying flavor characteristics with simple, fast, and nondestructive sample preparation. E-nose was able to provide an overview of the odor features of the samples. It could clearly distinguish the changes in flavor characteristics produced by *L. plantarum* under capsaicin stress. In parallel, GC–IMS could spot the specific VOCs conveying the overall response, such as ethyl acetate, acetic acid, 2-heptanone, 1-penten-3-one, and 3-methyl-3-buten-1-ol. Overall, the aroma-producing property of *L. plantarum* could be affected by capsaicin stress. Therefore, the present work could provide a basis for investigating the aroma-producing property of *L. plantarum* and shed light on establishing more comprehensive and rapid methods for identifying flavor characteristics.

## Figures and Tables

**Table 1:** Peak intensity of VOCs produced by *L. plantarum* fermentation for 24 h under different capsaicin concentrations were characterized by GC–IMS (mean ± sd).

Compounds	CAS	Formula	RI *	RT [s]	DT [ms]	Peak Intensity (V)			
						A	B	C	D
Alcohols (12)									
1-Propanethiol	107-03-9	C_3_H_8_S	823.2	264.886	1.17068	5.61 × 10^2^ ± 0.75 *^a #^*	3.50 × 10^2^ ± 8.97 *^c^*	3.88 × 10^2^ ± 10.80 *^b^*	3.18 × 10^2^ ± 7.57 *^d^*
2-Butoxyethanol	111-76-2	C_6_H_14_O_2_	885.3	290.249	1.20111	1.80 × 10^2^ ± 34.80 *^a^*	1.20 × 10^2^ ± 15.60 ^*b*^	1.37 × 10^2^ ± 5.08 ^*ab*^	1.32 × 10^2^ ± 7.13 *^ab^*
2-Furanmethanethiol	98-02-2	C_5_H_6_OS	895.6	294.418	1.34532	1.70 × 10^2^ ± 38.60 ^*ab*^	2.01 × 10^2^ ± 22.50 ^*a*^	1.38 × 10^2^ ± 8.64 ^*ab*^	1.21 × 10^2^ ± 16.00 ^*b*^
2-Hexanol	626-93-7	C_6_H_14_O	808.7	258.979	1.28001	2.21 × 10^2^ ± 31.80 ^*a*^	1.21 × 10^2^ ± 3.22 ^*c*^	1.69 × 10^2^ ± 2.43 ^*b*^	1.44 × 10^2^ ± 3.62 ^*b*^
2-Methyl-1-butanol	137-32-6	C_5_H_12_O	724.9	224.785	1.22777	1.93 × 10^2^ ± 15.90 *^c^*	2.62 × 10^2^ ± 16.60 ^*b*^	4.22 × 10^2^ ± 10.50 *^a^*	4.64 × 10^2^ ± 31.10 ^*a*^
3-Methyl-1-butanol-D	123-51-3	C_5_H_12_O	1178.6	760.516	1.48999	2.70 × 10^3^ ± 25.00 *^b^*	2.73 × 10^3^ ± 47.50 ^*b*^	3.03 × 10^3^ ± 22.70 ^*a*^	3.32 × 10^3^ ± 1.38 × 10^2 *a*^
3-Methyl-1-butanol-M	123-51-3	C_5_H_12_O	1178	757.997	1.24036	4.02 × 10^3^ ± 2.49 × 10^2^ *^a^*	2.61 × 10^3^ ± 88.30 *^b^*	2.61 × 10^3^ ± 50.10 ^*b*^	2.63 × 10^3^ ± 1.24 × 10^2^ ^*b*^
3-Methyl-3-buten-1-ol	763-32-6	C_5_H_10_O	1258.7	1067.888	1.15898	2.74 × 10^2^ ± 8.59 *^b^*	3.00 × 10^2^ ± 28.80 *^ab^*	3.36 × 10^2^ ± 34.00 *^ab^*	3.61 × 10^2^ ± 22.10 ^*a*^
5-Methylfurfuryl alcohol	3857-25-8	C_6_H_8_O_2_	953.8	326.696	1.2638	1.36 × 10^2^ ± 8.00 ^*a*^	1.57 × 10^2^ ± 9.41 *^a^*	1.52 × 10^2^ ± 5.46 *^a^*	1.49 × 10^2^ ± 7.48 *^a^*
*cis*-3-Hexen-1-ol	928-96-1	C_6_H_12_O	822.6	264.628	1.22689	1.63 × 10^2^ ± 5.99 ^*a*^	75.60 ± 1.41 ^*c*^	1.01 × 10^2^ ± 2.67 ^*b*^	73.00 ± 1.15 ^*c*^
Ethanol	64-17-5	C_2_H_6_O	887.7	291.195	1.13063	1.27 × 10^2^ ± 13.10 ^*a*^	79.80 ± 13.30 ^*b*^	1.11 × 10^2^ ± 1.37 *^a^*	1.12 × 10^2^ ± 12.40 ^*a*^
Isobutanol	78-83-1	C_4_H_10_O	1077.9	475.544	1.17179	7.75 × 10^2^ ± 52.10 ^*a*^	4.78 × 10^2^ ± 11.70 ^*bc*^	4.63 × 10^2^ ± 17.40 ^*c*^	5.14 × 10^2^ ± 16.80 ^*b*^
Esters (11)									
2-Methylbutyl acetate	624-41-9	C_7_H_14_O_2_	895.2	294.269	1.29296	1.56 × 10^2^ ± 8.78 *^c^*	2.44 × 10^2^ ± 6.28 *^a^*	1.97 × 10^2^ ± 3.12 *^b^*	1.77 × 10^2^ ± 11.00 *^bc^*
Diethyl malonate	105-53-3	C_7_H_12_O_4_	1077.9	475.544	1.25078	1.22 × 10^3^ ± 46.50 *^a^*	1.02 × 10^3^ ± 22.20 *^a^*	1.07 × 10^3^ ± 97.60 *^a^*	1.04 × 10^3^ ± 33.60 *^a^*
Ethyl 2-methylbutyrate	7452-79-1	C_7_H_14_O_2_	1014.3	383.166	1.228	1.79 × 10^2^ ± 18.30 *^a^*	2.06 × 10^2^ ± 7.20 *^a^*	1.87 × 10^2^ ± 5.33 *^a^*	1.86 × 10^2^ ± 8.86 *^a^*
Ethyl 3-hydroxybutyrate	5405-41-4	C_6_H_12_O_3_	901.6	297.442	1.16822	82.40 ± 50.90 *^a^*	92.90 ± 20.10 *^a^*	1.10 × 10^2^ ± 4.76 *^a^*	99.80 ± 24.80 *^a^*
Ethyl acetate	141-78-6	C_4_H_8_O_2_	880.9	288.431	1.33717	4.36 × 10^3^ ± 2.24 × 10^2 *a*^	3.92 × 10^3^ ± 48.00 *^ab^*	3.59 × 10^3^ ± 64.00 *^bc^*	3.47 × 10^3^ ± 93.50 *^c^*
Ethyl crotonate	623-70-1	C_6_H_10_O_2_	1140.8	632.689	1.1866	96.70 ± 6.44 *^b^*	1.09 × 10^2^ ± 2.09 *^ab^*	1.14 × 10^2^ ± 6.65 *^a^*	1.13 × 10^2^ ± 6.94 *^a^*
Ethyl pentanoate	539-82-2	C_7_H_14_O_2_	907.7	300.837	1.26713	3.10 × 10^2^ ± 94.20 *^a^*	3.27 × 10^2^ ± 38.80 *^a^*	3.07 × 10^2^ ± 19.20 *^a^*	3.00 × 10^2^ ± 19.10 *^a^*
Isopropyl acetate	108-21-4	C_5_H_10_O_2_	907	300.471	1.15882	6.87 × 10^2^ ± 1.15 × 10^2^ *^a^*	3.55 × 10^2^ ± 25.30 *^b^*	3.13 × 10^2^ ± 7.05 *^b^*	3.03 × 10^2^ ± 26.70 *^b^*
Methyl acetate	79-20-9	C_3_H_6_O_2_	810.2	259.584	1.19708	2.30 × 10^2^ ± 2.32 *^a^*	1.30 × 10^2^ ± 7.11 *^c^*	1.53 × 10^2^ ± 3.91 *^b^*	1.35 × 10^2^ ± 1.91 *^c^*
Methyl isovalerate	556-24-1	C_6_H_12_O_2_	1028	400.946	1.1944	3.51 × 10^2^ ± 16.00 *^b^*	3.88 × 10^2^ ± 5.63 *^a^*	3.61 × 10^2^ ± 3.68 *^ab^*	3.52 × 10^2^ ± 7.84 *^ab^*
gamma-Octalactone	104-50-7	C_8_H_14_O_2_	1245.8	1023.798	1.33273	4.06 × 10^2^ ± 5.65 ^*a*^	2.93 × 10^2^ ± 16.60 ^*b*^	4.08 × 10^2^ ± 78.30 ^*a*^	3.70 × 10^2^ ± 15.00 ^*a*^
Ketones (8)									
1-Penten-3-one	1629-58-9	C_5_H_8_O	1026.1	398.551	1.31696	94.90 ± 6.23 *^c^*	1.42 × 10^2^ ± 6.94 *^ab^*	1.55 × 10^2^ ± 4.33 *^a^*	1.38 × 10^2^ ± 6.71 *^b^*
2-Butanone	78-93-3	C_4_H_8_O	898.5	295.719	1.24091	7.44 × 10^2^ ± 53.70 *^b^*	9.15 × 10^2^ ± 38.90 *^a^*	7.70 × 10^2^ ± 5.81 *^ab^*	7.96 × 10^2^ ± 61.40 *^ab^*
2-Cyclohexen-1-one	930-68-7	C_6_H_8_O	914.4	304.625	1.40015	3.34 × 10^3^ ± 2.33 × 10^2 *a*^	2.18 × 10^3^ ± 21.80 *^c^*	2.43 × 10^3^ ± 27.50 *^b^*	2.39 × 10^3^ ± 50.10 *^b^*
2-Heptanone	110-43-0	C_7_H_14_O	1158.5	681.077	1.26393	5.60 × 10^2^ ± 28.7 *^b^*	6.66 × 10^2^ ± 19.80 *^a^*	6.28 × 10^2^ ± 6.66 *^a^*	6.37 × 10^2^ ± 18.80 *^a^*
2-Pentanone	107-87-9	C_5_H_10_O	982.7	342.913	1.36883	1.49 × 10^2^ ± 7.48 *^c^*	3.09 × 10^2^ ± 18.90 *^a^*	2.75 × 10^2^ ± 11.90 *^ab^*	2.62 × 10^2^ ± 13.80 *^b^*
Acetoin	513-86-0	C_4_H_8_O_2_	724.8	224.762	1.0636	4.10 × 10^2^ ± 10.60 *^a^*	3.08 × 10^2^ ± 61.10 *^a^*	4.00 × 10^2^ ± 17.60 *^a^*	3.81 × 10^2^ ± 54.10 *^a^*
(-)-Carvone	6485-40-1	C_10_H_14_O	1179.8	765.243	1.3148	1.17 × 10^3^ ± 52.20 *^a^*	1.08 × 10^3^ ± 17.50 ^*ab*^	1.00 × 10^3^ ± 51.30 ^*b*^	1.01 × 10^3^ ± 33.30 ^*b*^
Isophorone	78-59-1	C_9_H_14_O	1119.9	580.59	1.25872	2.63 × 10^2^ ± 25.20 *^a^*	1.95 × 10^2^ ± 7.17 *^b^*	1.88 × 10^2^ ± 10.60 *^b^*	1.75 × 10^2^ ± 12.20 *^b^*
Acids (4)									
2-Methylbutanoic acid	116-53-0	C_5_H_10_O_2_	872.8	285.138	1.20659	1.38 × 10^2^ ± 14.50 *^a^*	1.11 × 10^2^ ± 5.56 *^ab^*	1.01 × 10^2^ ± 7.51 *^bc^*	92.80 ± 3.27 *^c^*
Acetic acid	64-19-7	C_2_H_4_O_2_	1439.7	1686.517	1.15796	7.13 × 10^3^ ± 1.81 × 10^2 *a*^	7.22 × 10^3^ ± 4.79 × 10^2^ *^a^*	7.73 × 10^3^ ± 1.07 × 10^3^ ^*a*^	7.65 × 103 ± 5.08 × 10^2^ *^a^*
Heptanoic acid	111-14-8	C_7_H_14_O_2_	1073.3	464.167	1.36185	4.64 × 10^2^ ± 2.04 × 10^2 *a*^	2.96 × 10^2^ ± 74.00 *^a^*	1.05 × 10^3^ ± 6.92 × 10^2 *a*^	3.28 × 10^2^ ± 8.83 *^a^*
Isobutyric acid	79-31-2	C_4_H_8_O_2_	729	226.443	1.15617	59.20 ± 25.90 *^b^*	1.42 × 10^2^ ± 39.70 *^a^*	1.36 × 10^2^ ± 9.65 *^ab^*	1.56 × 10^2^ ± 42.00 *^a^*
Aldehydes (4)									
4-Methoxybenzaldehyde	123-11-5	C_8_H_8_O_2_	1245.4	1022.538	1.20737	6.61 × 10^2^ ± 21.60 *^a^*	5.73 × 10^2^ ± 15.80 *^b^*	7.55 × 10^2^ ± 65.80 *^a^*	6.80 × 10^2^ ± 56.90 *^a^*
Acrolein	107-02-8	C_3_H_4_O	823.8	265.156	1.06297	3.55 × 10^2^ ± 77.30 *^a^*	1.33 × 10^2^ ± 6.91 *^b^*	1.31 × 10^2^ ± 4.85 × 10^−1 *b*^	1.12 × 10^2^ ± 7.26 *^b^*
Butanal	123-72-8	C_4_H_8_O	882.4	289.035	1.0965	6.75 × 10^2^ ± 1.16 × 10^2^ ^*a*^	3.35 × 10^2^ ± 3.37 *^b^*	3.20 × 10^2^ ± 6.24 *^b^*	3.14 × 10^2^ ± 19.70 *^b^*
*trans*-2-Pentenal	1576-87-0	C_5_H_8_O	1157.6	677.582	1.35994	4.87 × 10^2^ ± 18.70 *^b^*	9.03 × 10^2^ ± 31.50 *^a^*	8.63 × 10^2^ ± 28.80 *^a^*	8.42 × 10^2^ ± 40.30 *^a^*
Others (9)									
1,1-Diethoxyethane	105-57-7	C_6_H_14_O_2_	738.4	230.277	1.13101	1.95 × 10^2^ ± 1.07 × 10^2 *a*^	3.20 × 10^2^ ± 82.00 *^a^*	3.89 × 10^2^ ± 11.70 *^a^*	3.91 × 10^2^ ± 22.30 *^a^*
2,2,4,6,6-Pentamethylheptane	13475-82-6	C_12_H_26_	917.4	306.324	1.0399	1.26 × 10^2^ ± 7.43 *^a^*	72.00 ± 3.01 *^b^*	59.00 ± 3.10 ^*bc*^	55.10 ± 4.93 ^*c*^
2-Ethyl-3,5-dimethylpyrazine	13925-07-0	C_8_H_12_N_2_	1083.3	489.054	1.21129	1.66 × 10^2^ ± 3.07 ^*a*^	1.76 × 10^2^ ± 2.54 ^*a*^	1.27 × 10^2^ ± 4.79 *^c^*	1.43 × 10^2^ ± 5.97 *^b^*
2-sec-Butyl-3-methoxypyrazine	24168-70-5	C_9_H_14_N_2_O	1077.9	475.467	1.28604	2.78 × 102 ± 41.80 *^a^*	2.98 × 102 ± 4.14 *^a^*	3.57 × 102 ± 72.70 *^a^*	3.16 × 102 ± 7.87 *^a^*
alpha-Pinene	80-56-8	C_10_H_16_	1003.7	369.402	1.28369	80.00 ± 9.55 *^a^*	85.70 ± 4.26 ^*a*^	80.10 ± 2.57 ^*a*^	89.00 ± 4.30 ^*a*^
beta-Myrcene	123-35-3	C_10_H_16_	985	345.005	1.21594	9.39 × 10^2^ ± 41.60 *^ab^*	9.80 × 10^2^ ± 69.40 ^*a*^	8.96 × 10^2^ ± 9.06 ^*ab*^	8.58 × 10^2^ ± 26.00 ^*b*^
beta-Pinene	127-91-3	C_10_H_16_	1107.2	548.783	1.29583	2.42 × 10^2^ ± 15.40 *^b^*	2.88 × 10^2^ ± 10.10 *^a^*	3.00 × 10^2^ ± 5.83 *^a^*	3.16 × 10^2^ ± 13.20 *^a^*
Decalin	91-17-8	C_10_H_18_	1052.4	432.721	1.26618	72.50 ± 5.85 *^ab^*	62.50 ± 4.89 *^b^*	78.50 ± 6.92 *^a^*	80.20 ± 6.37 *^a^*
N-Nitrosomorpholine	59-89-2	C_4_H_8_N_2_O_2_	1106.9	548.072	1.19339	2.13 × 10^2^ ± 7.97 *^ab^*	2.21 × 10^2^ ± 4.52 *^ab^*	2.08 × 10^2^ ± 4.96 *^b^*	2.31 × 10^2^ ± 5.36 *^a^*

* RI, RT, and Dt stand for retention index, retention time, and drift time, respectively. ^#^ For each molecule, sd values followed by a common superscript identify no significant differences.

**Table 2:** Peak intensities of VOCs of *L. plantarum* along fermentation under capsaicin stress were characterized by GC–IMS (mean ± sd).

Compounds	Peak Intensity				
	BT00	BT06	BT12	BT18	BT24
Alcohols (12)					
1-Propanethiol	4.36 × 10^2^ ± 4.74 *^c^* *	3.79 × 10^2^ ± 9.26 ^*d*^	5.03 × 10^2^ ± 5.39 *^b^*	5.19 × 10^2^ ± 8.35 *^b^*	6.15 × 10^2^ ± 16.40 ^*a*^
2-Butoxyethanol	3.35 × 10^2^ ± 6.14 ^*a*^	2.66 × 10^2^ ± 14.60 ^*a*^	2.70 × 10^2^ ± 4.12 *^a^*	2.71 × 10^2^ ± 5.34 ^*a*^	2.63 × 10^2^ ± 1.00 × 10^2 *a*^
2-Furanmethanethiol	95.50 ± 3.04 *^c^*	1.08 × 10^2^ ± 3.51 ^*bc*^	2.32 × 10^2^ ± 19.00 ^*a*^	1.78 × 10^2^ ± 3.53 ^*ab*^	3.19 × 10^2^ ± 1.66 × 10^2^ ^*a*^
2-Hexanol	7.23 × 10^2^ ± 10.50 ^*a*^	5.05 × 10^2^ ± 19.10 ^*b*^	2.14 × 10^2^ ± 2.08 ^*cd*^	1.84 × 10^2^ ± 1.48 ^*d*^	2.68 × 10^2^ ± 76.00 ^*c*^
2-Methyl-1-butanol	1.32 × 10^3^ ± 41.30 ^*a*^	8.77 × 10^2^ ± 24.60 ^*b*^	7.35 × 10^2^ ± 11.80 ^*c*^	7.58 × 10^2^ ± 14.10 ^*c*^	4.09 × 10^2^ ± 41.40 ^*d*^
3-Methyl-1-butanol-D	4.29 × 10^3^ ± 18.10 *^ab^*	4.46 × 10^3^ ± 87.90 ^*a*^	4.35 × 10^3^ ± 29.40 ^*ab*^	4.20 × 10^3^ ± 45.70 ^*ab*^	4.12 × 10^3^ ± 2.35 × 10^2 *b*^
3-Methyl-1-butanol-M	3.94 × 10^3^ ± 37.40 ^*ab*^	4.05 × 10^3^ ± 1.84×10^2 *a*^	3.72 × 10^3^ ± 59.40 ^*ab*^	3.48 × 10^3^ ± 63.10 ^*b*^	4.18 × 10^3^ ± 8.06 × 10^2 *a*^
3-Methyl-3-buten-1-ol	6.17 × 10^2^ ± 44.50 ^*ab*^	5.75 × 10^2^ ± 73.80 ^*b*^	6.44 × 10^2^ ± 35.70 ^*ab*^	8.53 × 10^2^ ± 59.20 ^*a*^	8.44 × 10^2^ ± 1.13 × 10^2 *a*^
5-Methylfurfuryl alcohol	2.20 × 10^2^ ± 11.40 ^*c*^	2.26 × 10^2^ ± 2.21 ^*bc*^	2.54 × 10^2^ ± 5.41 ^*ab*^	2.74 × 10^2^ ± 2.80 ^*a*^	2.31 × 10^2^ ± 13.90 ^*bc*^
*cis*-3-Hexen-1-ol	1.26 × 10^2^ ± 3.88 ^*a*^	88.40 ± 5.13 ^*c*^	1.02 × 10^2^ ± 2.14 ^*b*^	1.12 × 10^2^ ± 1.86 ^*ab*^	1.30 × 10^2^ ± 12.60 ^*a*^
Ethanol	3.67 × 10^2^ ± 18.60 ^*a*^	3.01 × 10^2^ ± 16.00 ^*a*^	2.53 × 10^2^ ± 1.91 ^*a*^	2.31 × 10^2^ ± 11.10 *^a^*	2.27 × 10^2^ ± 1.18 × 10^2 *a*^
Isobutanol	5.71 × 10^2^ ± 11.90 ^*b*^	6.73 × 10^2^ ± 6.40 ^*a*^	6.89 × 10^2^ ± 15.20 ^*a*^	6.63 × 10^2^ ± 20.50 ^*a*^	8.00 × 10^2^ ± 1.52 × 10^2 *a*^
Esters (11)					
2-Methylbutyl acetate	99.50 ± 4.49 ^*e*^	1.53 × 10^2^ ± 2.90 ^*d*^	2.94 × 10^2^ ± 5.46 ^*c*^	3.71 × 10^2^ ± 6.05 ^*a*^	3.39 × 10^2^ ± 9.33 ^*b*^
Diethyl malonate	1.20 × 10^3^ ± 24.30 ^*b*^	1.65 × 10^3^ ± 2.31×10^2 *a*^	1.62 × 10^3^ ± 29.60 ^*a*^	1.59 × 10^3^ ± 37.00 ^*a*^	1.57 × 10^3^ ± 64.10 ^*a*^
Ethyl 2-methylbutyrate	3.56 × 10^2^ ± 5.43 ^*a*^	3.10 × 10^2^ ± 5.20 ^*b*^	3.06 × 10^2^ ± 18.70 ^*bc*^	3.14 × 10^2^ ± 5.72 ^*ab*^	2.81 × 10^2^ ± 8.99 ^*c*^
Ethyl 3-hydroxybutyrate	1.86 × 10^2^ ± 22.00 ^*a*^	2.04 × 10^2^ ± 19.10 ^*a*^	1.95 × 10^2^ ± 9.19 *^a^*	1.96 × 10^2^ ± 1.45 ^*a*^	1.64 × 10^2^ ± 1.07 × 10^2 *a*^
Ethyl acetate	3.01 × 10^3^ ± 18.20 ^*d*^	4.16 × 10^3^ ± 60.70 ^*c*^	5.46 × 10^3^ ± 53.40 ^*b*^	5.94 × 10^3^ ± 45.70 ^*ab*^	6.66 × 10^3^ ± 1.02 × 10^3 *a*^
Ethyl crotonate	2.27 × 10^2^ ± 3.85 ^*a*^	1.89 × 10^2^ ± 3.33 ^*a*^	1.99 × 10^2^ ± 19.40 ^*a*^	1.95 × 10^2^ ± 8.02 ^*a*^	1.61 × 10^2^ ± 13.20 ^*b*^
Ethyl pentanoate	5.82 × 10^2^ ± 24.40 ^*a*^	5.89 × 10^2^ ± 30.70 ^*a*^	5.83 × 10^2^ ± 2.94 *^a^*	4.52 × 10^2^ ± 16.70 *^a^*	5.17 × 10^2^ ± 1.81 × 10^2 *a*^
Isopropyl acetate	3.94 × 10^2^ ± 9.07 ^*b*^	4.85 × 10^2^ ± 40.50 ^*a*^	4.51 × 10^2^ ± 4.14 ^*ab*^	4.42 × 10^2^ ± 5.51 ^*ab*^	5.30 × 10^2^ ± 1.26 × 10^2 *a*^
Methyl acetate	4.22 × 10^2^ ± 3.88 ^*a*^	3.54 × 10^2^ ± 19.50 ^*b*^	2.38 × 10^2^ ± 6.10 ^*c*^	1.57 × 10^2^ ± 4.21 ^*d*^	2.74 × 10^2^ ± 35.40 ^*c*^
Methyl isovalerate	6.45 × 10^2^ ± 1.81 ^*a*^	6.52 × 10^2^ ± 17.70 ^*a*^	6.49 × 10^2^ ± 5.72 *^a^*	6.63 × 10^2^ ± 8.82 ^*a*^	6.32 × 10^2^ ± 48.60 ^*a*^
gamma-Octalactone	6.73 × 10^2^ ± 19.20 ^*a*^	8.14 × 10^2^ ± 53.80 ^*a*^	4.18 × 10^2^ ± 18.90 ^*b*^	4.58 × 10^2^ ± 32.50 ^*b*^	4.51 × 10^2^ ± 69.60 ^*b*^
Ketones (8)					
1-Penten-3-one	1.77 × 10^2^ ± 4.43 ^*c*^	2.47 × 10^2^ ± 1.43 *^a^*	2.55 × 10^2^ ± 4.85 ^*a*^	2.70 × 10^2^ ± 9.97 *^a^*	1.95 × 10^2^ ± 9.25 ^*b*^
2-Butanone	1.69 × 10^3^ ± 82.20 ^*a*^	1.63 × 10^3^ ± 74.30 *^a^*	1.55 × 10^3^ ± 26.40 ^*a*^	1.57 × 10^3^ ± 15.10 *^a^*	1.26 × 10^3^ ± 3.08 × 10^2 *a*^
2-Cyclohexen-1-one	5.83 × 10^3^ ± 54.10 ^*a*^	4.57 × 10^3^ ± 1.72 × 10^2 *b*^	3.77 × 10^3^ ± 24.00 ^*c*^	3.55 × 10^3^ ± 21.40 ^*d*^	4.16 × 10^3^ ± 3.55 × 10^2 *bc*^
2-Heptanone	1.89 × 10^2^ ± 5.28 ^*d*^	3.49 × 10^2^ ± 11.50 *^c^*	1.02 × 10^3^ ± 11.90 ^*ab*^	1.04 × 10^3^ ± 19.20 ^*a*^	9.86 × 10^2^ ± 32.50 ^*b*^
2-Pentanone	78.90 ± 5.19 *^d^*	1.14 × 10^2^ ± 17.30 ^*c*^	3.75 × 10^2^ ± 8.37 ^*b*^	5.17 × 10^2^ ± 7.64 ^*a*^	4.01 × 10^2^ ± 34.50 ^*b*^
Acetoin	7.22 × 10^2^ ± 1.20 × 10^2 *a*^	5.34 × 10^2^ ± 28.00 ^*a*^	4.46 × 10^2^ ± 7.64 ^*a*^	4.02 × 10^2^ ± 9.21 ^*a*^	4.80 × 10^2^ ± 2.91 × 10^2 *a*^
(-)-Carvone	1.92 × 10^3^ ± 14.90 ^*a*^	1.80 × 10^3^ ± 44.90 ^*a*^	1.67 × 10^3^ ± 64.70 ^*a*^	1.32 × 10^3^ ± 59.60 ^*b*^	1.66 × 10^3^ ± 1.96 × 10^2 *a*^
Isophorone	2.59 × 10^2^ ± 5.55 ^*a*^	3.10 × 10^2^ ± 15.90 ^*a*^	2.87 × 10^2^ ± 10.30 *^a^*	2.65 × 10^2^ ± 13.40 ^*a*^	2.76 × 10^2^ ± 35.90 ^*a*^
Acids (4)					
2-Methylbutanoic acid	2.26 × 10^2^ ± 3.97 ^*a*^	1.84 × 10^2^ ± 12.00 ^*b*^	1.66 × 10^2^ ± 8.23 ^*b*^	1.18 × 10^2^ ± 1.07 ^*c*^	1.68 × 10^2^ ± 8.39 ^*b*^
Acetic acid	8.18 × 10^3^ ± 1.21 × ×10^3 *c*^	9.50 × 10^3^ ± 1.32 × 10^3 *bc*^	1.30 × 10^4^ ± 4.87 × 10^2 *ab*^	1.79 × 10^4^ ± 1.67 × 10^3 *a*^	1.56 × 10^4^ ± 4.01 × 10^3 *ab*^
Heptanoic acid	6.40 × 10^2^ ± 1.37 × 10^2 *a*^	1.31 × 10^3^ ± 1.19 × 10^3 *a*^	8.24 × 10^2^ ± 1.48 × 10^2 *a*^	1.38 × 10^3^ ± 4.92 × 10^2 *a*^	8.44 × 10^2^ ± 5.76 × 10^2 *a*^
Isobutyric acid	3.24 × 10^2^ ± 1.08 × 10^2 *a*^	4.06 × 10^2^ ± 12.50 ^*a*^	4.18 × 10^2^ ± 9.14 ^*a*^	4.22 × 10^2^ ± 12.00 ^*a*^	2.95 × 10^2^ ± 1.83 × 10^2 *a*^
Aldehydes (4)					
4-Methoxybenzaldehyde	1.36 × 10^3^ ± 13.30 ^*a*^	1.47 × 10^3^ ± 86.10 ^*a*^	9.64 × 10^2^ ± 5.35 ^*b*^	1.11 × 10^3^ ± 79.50 ^*ab*^	9.68 × 10^2^ ± 1.91 × 10^2 *b*^
Acrolein	1.58 × 10^2^ ± 2.13 ^*ab*^	1.39 × 10^2^ ± 14.60 ^*b*^	1.52 × 10^2^ ± 3.96 ^*ab*^	1.52 × 10^2^ ± 1.42 ^*ab*^	2.34 × 10^2^ ± 95.90 *^a^*
Butanal	5.14 × 10^2^ ± 3.89 ^*a*^	5.57 × 10^2^ ± 36.30 *^a^*	4.70 × 10^2^ ± 8.23 ^*ab*^	4.53 × 10^2^ ± 1.89 ^*b*^	5.78 × 10^2^ ± 1.59 × 10^2 *a*^
*trans*-2-Pentenal	2.45 × 10^2^ ± 7.56 ^*d*^	4.98 × 10^2^ ± 12.80 ^*c*^	1.45 × 10^3^ ± 14.10 ^*a*^	1.56 × 10^3^ ± 34.20 ^*a*^	1.28 × 10^3^ ± 1.11 × 10^2 *b*^
Others (9)					
1,1-Diethoxyethane	6.60 × 10^2^ ± 39.00 ^*a*^	7.59 × 10^2^ ± 46.30 ^*a*^	8.17 × 10^2^ ± 17.00 ^*a*^	9.00 × 10^2^ ± 29.80 ^*a*^	7.05 × 10^2^ ± 5.52 × 10^2 *a*^
2,2,4,6,6-Pentamethylheptane	57.30 ± 2.16 *^c^*	86.20 ± 6.63 ^*ab*^	95.70 ± 5.15 ^*a*^	70.30 ± 3.35 ^*b*^	1.10 × 10^2^ ± 15.90 ^*a*^
2-Ethyl-3,5-dimethylpyrazine	3.05 × 10^2^ ± 11.90 ^*a*^	2.95 × 10^2^ ± 5.52 ^*a*^	2.87 × 10^2^ ± 6.83 *^a^*	3.03 × 10^2^ ± 2.63 ^*a*^	2.97 × 10^2^ ± 39.60 ^*a*^
2-sec-Butyl-3-methoxypyrazine	4.86 × 10^2^ ± 9.69 ^*a*^	5.62 × 10^2^ ± 1.32 × 10^2 *a*^	5.23 × 10^2^ ± 11.50 ^*a*^	5.45 × 10^2^ ± 33.70 ^*a*^	4.58 × 10^2^ ± 72.40 ^*a*^
alpha-Pinene	1.86 × 10^2^ ± 2.32 ^*a*^	1.50 × 10^2^ ± 5.47 ^*b*^	1.42 × 10^2^ ± 2.67 ^*bc*^	1.57 × 10^2^ ± 9.29 ^*b*^	1.27 × 10^2^ ± 7.95 ^*c*^
beta-Myrcene	1.16 × 10^3^ ± 74.90 ^*b*^	1.12 × 10^3^ ± 24.70 *^b^*	1.36 × 10^3^ ± 18.60 ^*a*^	1.47 × 10^3^ ± 20.60 *^a^*	1.47 × 10^3^ ± 15.30 ^*a*^
beta-Pinene	5.81 × 10^2^ ± 11.00 *^a^*	5.04 × 10^2^ ± 13.80 ^*b*^	4.94 × 10^2^ ± 6.52 ^*b*^	5.17 × 10^2^ ± 15.40 ^*b*^	4.24 × 10^2^ ± 4.26 ^*c*^
Decalin	2.03 × 10^2^ ± 3.87 ^*a*^	1.65 × 10^2^ ± 10.40 ^*b*^	96.90 ± 3.18 ^*c*^	95.30 ± 2.61 *^c^*	99.80 ± 12.70 ^*c*^
N-Nitrosomorpholine	4.72 × 10^2^ ± 7.53 *^a^*	3.78 × 10^2^ ± 15.50 ^*b*^	3.62 × 10^2^ ± 12.90 ^*b*^	3.80 × 10^2^ ± 2.35 ^*b*^	3.91 × 10^2^ ± 24.50 *^b^*

* For each VOC, sd values followed by a common superscript identify no significant differences.

**Table 3:** Performance description of the E-nose sensors.

Sensor Number	Sensor Name	Performance Description
1	LY2/LG	Sensitive to oxidizing gas
2	LY2/G	Sensitive to ammonia, carbon monoxide
3	LY2/AA	Sensitive to ethanol
4	LY2/Gh	Sensitive to ammonia/organic amines
5	LY2/gCT1	Sensitive to hydrogen sulfide
6	LY2/gCT	Sensitive to propane/butane
7	T30/1	Sensitive to organic solvents
8	P10/1	Sensitive to hydrocarbons
9	P10/2	Sensitive to methane
10	P40/1	Sensitive to fluorine
11	T70/2	Sensitive to aromatic compounds
12	PA/2	Sensitive to ethanol, ammonia/organic amines
13	P30/1	Sensitive to polar compounds (ethanol)
14	P40/2	Sensitive to heteroatom/chloride/aldehydes
15	P30/2	Sensitive to alcohol
16	T40/2	Sensitive to aldehydes
17	T40/1	Sensitive to chlorinated compounds
18	TA/2	Sensitive to air quality

## Data Availability

The data presented in this study are available on request from the corresponding author.

## Supplementary Materials

The following supporting information can be downloaded at: https://www.mdpi.com/article/10.3390/molecules29010107/s1, Table S1: Response values (mean ± sd) of the E-nose to *L. plantarum* fermentation for 24 h under different capsaicin concentrations.; Table S2: Response values (mean ± sd) of the E-nose to *L. plantarum* along fermentation under capsaicin stress.

## References

[1] Heng Z., Xu X., Xu X., Li Y., Wang H., Huang W., Yan S., Li T. (2023). Integrated Transcriptomic and Metabolomic Analysis of Chili Pepper Fruits Provides New Insight into the Regulation of the Branched Chain Esters and Capsaicin Biosynthesis. Food Res. Int..

[2] Li M., Bao X., Zhang X., Ren H., Cai S., Hu X., Yi J. (2022). Exploring the Phytochemicals and Inhibitory Effects against α-Glucosidase and Dipeptidyl Peptidase-IV in Chinese Pickled Chili Pepper: Insights into Mechanisms by Molecular Docking Analysis. LWT.

[3] Sanatombi K. (2023). Antioxidant Potential and Factors Influencing the Content of Antioxidant Compounds of Pepper: A Review with Current Knowledge. Compr. Rev. Food Sci. Food Saf..

[4] Xu X., Wu B., Zhao W., Lao F., Chen F., Liao X., Wu J. (2021). Shifts in Autochthonous Microbial Diversity and Volatile Metabolites during the Fermentation of Chili Pepper (*Capsicum frutescens* L.). Food Chem..

[5] Li Z., Dong L., Jeon J., Kwon S.Y., Zhao C., Baek H.H. (2019). Characterization and Evaluation of Aroma Quality in Doubanjiang, a Chinese Traditional Fermented Red Pepper Paste, Using Aroma Extract Dilution Analysis and a Sensory Profile. Molecules.

[6] Shi Q., Tang H., Mei Y., Chen J., Wang X., Liu B., Cai Y., Zhao N., Yang M., Li H. (2023). Effects of Endogenous Capsaicin Stress and Fermentation Time on the Microbial Succession and Flavor Compounds of Chili Paste (a Chinese Fermented Chili Pepper). Food Res. Int..

[7] Ye Z., Shang Z., Li M., Zhang X., Ren H., Hu X., Yi J. (2022). Effect of Ripening and Variety on the Physiochemical Quality and Flavor of Fermented Chinese Chili Pepper (Paojiao). Food Chem..

[8] Li Y., Luo X., Long F., Wu Y., Zhong K., Bu Q., Huang Y., Gao H. (2023). Quality Improvement of Fermented Chili Pepper by Inoculation of Pediococcus Ethanolidurans M1117: Insight into Relevance of Bacterial Community Succession and Metabolic Profile. LWT.

[9] Chen Y., Li P., Liao L., Qin Y., Jiang L., Liu Y. (2021). Characteristic Fingerprints and Volatile Flavor Compound Variations in Liuyang Douchi during Fermentation via HS-GC-IMS and HS-SPME-GC-MS. Food Chem..

[10] Hu Y., Zhang L., Wen R., Chen Q., Kong B. (2020). Role of Lactic Acid Bacteria in Flavor Development in Traditional Chinese Fermented Foods: A Review. Crit. Rev. Food Sci. Nutr..

[11] López-Salas D., Oney-Montalvo J.E., Ramírez-Rivera E., Ramírez-Sucre M.O., Rodríguez-Buenfil I.M. (2022). Evaluation of the Volatile Composition and Sensory Behavior of Habanero Pepper during Lactic Acid Fermentation by *L. plantarum*. Foods.

[12] Peng S., Xu J., Xu J., Wang J., Zhang Y., Liao X., Zhao L. (2023). Microbial Community and Volatile Metabolites Related to the Fermentation Degree of Salted Fermented Chili Peppers. LWT.

[13] Duwat P., Ehrlich S.D., Gruss A. (1999). Effects of Metabolic Flux on Stress Response Pathways in Lactococcus Lactis. Mol. Microbiol..

[14] Yang H., He M., Wu C. (2021). Cross Protection of Lactic Acid Bacteria during Environmental Stresses: Stress Responses and Underlying Mechanisms. LWT.

[15] Gallegos J., Arce C., Jordano R., Arce L., Medina L.M. (2017). Target Identification of Volatile Metabolites to Allow the Differentiation of Lactic Acid Bacteria by Gas Chromatography-Ion Mobility Spectrometry. Food Chem..

[16] Wang J., Wei B.C., Wang X., Zhang Y., Gong Y.J. (2023). Aroma Profiles of Sweet Cherry Juice Fermented by Different Lactic Acid Bacteria Determined through Integrated Analysis of Electronic Nose and Gas Chromatography–Ion Mobility Spectrometry. Front. Microbiol..

[17] Yang C., Ye Z., Mao L., Zhang L., Zhang J., Ding W., Han J., Mao K. (2022). Analysis of Volatile Organic Compounds and Metabolites of Three Cultivars of Asparagus (*Asparagus officinalis* L.) Using E-Nose, GC-IMS, and LC-MS/MS. Bioengineered.

[18] Gao Y., Hou L., Gao J., Li D., Tian Z., Fan B., Wang F., Li S. (2021). Metabolomics Approaches for the Comprehensive Evaluation of Fermented Foods: A Review. Foods.

[19] Arnold¤ J.W., Senter S.D. (1998). Use of Digital Aroma Technology and SPME to Compare Volatile Compounds GC-MS Produced by Bacteria Isolated from Processed Poultry. J. Sci. Food Agric..

[20] Chen J.N., Zhang Y.Y., Huang X.H., Dong M., Dong X.P., Zhou D.Y., Zhu B.W., Qin L. (2023). Integrated Volatolomics and Metabolomics Analysis Reveals the Characteristic Flavor Formation in Chouguiyu, a Traditional Fermented Mandarin Fish of China. Food Chem..

[21] Zhao X., Feng J., Laghi L., Deng J., Dao X., Tang J., Ji L., Zhu C., Picone G. (2023). Characterization of Flavor Profile of “Nanx Wudl” Sour Meat Fermented from Goose and Pork Using Gas Chromatography–Ion Mobility Spectrometry (GC–IMS) Combined with Electronic Nose and Tongue. Foods.

[22] Ricci A., Cirlini M., Levante A., Dall’Asta C., Galaverna G., Lazzi C. (2018). Volatile Profile of Elderberry Juice: Effect of Lactic Acid Fermentation Using *L. plantarum*, *L. rhamnosus* and *L. casei* strains. Food Res. Int..

[23] Pang X.N., Chen C., Huang X.N., Yan Y.Z., Chen J.Y., Han B.Z. (2021). Influence of Indigenous Lactic Acid Bacteria on the Volatile Flavor Profile of Light-Flavor Baijiu. LWT.

[24] Gao T., Chen J., Xu J., Gu H., Zhao P., Wang W., Pan S., Tao Y., Wang H., Yang J. (2022). Screening of a Novel *Lactiplantibacillus plantarum* MMB-05 and *Lacticaseibacillus casei* Fermented Sandwich Seaweed Scraps: Chemical Composition, In Vitro Antioxidant, and Volatile Compounds Analysis by GC-IMS. Foods.

[25] Li X., Cheng X., Yang J., Wang X., Lü X. (2022). Unraveling the Difference in Physicochemical Properties, Sensory, and Volatile Profiles of Dry Chili Sauce and Traditional Fresh Dry Chili Sauce Fermented by *Lactobacillus plantarum* PC8 Using Electronic Nose and HS-SPME-GC-MS. Food Biosci..

[26] Lu Y., Tan X., Lv Y., Yang G., Chi Y., He Q. (2020). Flavor Volatiles Evolution of Chinese Horse Bean-Chili-Paste during Ripening, Accessed by GC×GC-TOF/MS and GC-MS-Olfactometry. Int. J. Food Prop..

[27] Chen C., Lu Y., Yu H., Chen Z., Tian H. (2019). Influence of 4 Lactic Acid Bacteria on the Flavor Profile of Fermented Apple Juice. Food Biosci..

[28] Wang Z., Dou R., Yang R., Cai K., Li C., Li W. (2021). Changes in Phenols, Polysaccharides and Volatile Profiles of Noni (*Morinda citrifolia* L.) Juice during Fermentation. Molecules.

[29] Chen H., Nie X., Peng T., Xiang L., Liu D., Luo H., Zhao Z. (2023). Effects of Low-Temperature and Low-Salt Fermentation on the Physicochemical Properties and Volatile Flavor Substances of Chinese Kohlrabi Using Gas Chromatography–Ion Mobility Spectrometry. Fermentation.

[30] Wang J., Wang R., Xiao Q., Liu C., Deng F., Zhou H. (2019). SPME/GC-MS Characterization of Volatile Compounds of Chinese Traditional-Chopped Pepper during Fermentation. Int. J. Food Prop..

[31] Gou Y., Ma X., Niu X., Ren X., Muhatai G., Xu Q. (2023). Exploring the Characteristic Aroma Components of Traditional Fermented Koumiss of Kazakh Ethnicity in Different Regions of Xinjiang by Combining Modern Instrumental Detection Technology with Multivariate Statistical Analysis Methods for Odor Activity Value and Sensory Analysis. Foods.

[32] Li C., Al-Dalali S., Wang Z., Xu B., Zhou H. (2022). Investigation of Volatile Flavor Compounds and Characterization of Aroma-Active Compounds of Water-Boiled Salted Duck Using GC–MS–O, GC–IMS, and E-Nose. Food Chem..

[33] Chen Y., Xu H., Ding S., Zhou H., Qin D., Deng F., Wang R. (2020). Changes in Volatile Compounds of Fermented Minced Pepper during Natural and Inoculated Fermentation Process Based on Headspace–Gas Chromatography–Ion Mobility Spectrometry. Food Sci. Nutr..

[34] Hu Y., Zhang L., Liu Q., Wang Y., Chen Q., Kong B. (2020). The Potential Correlation between Bacterial Diversity and the Characteristic Volatile Flavour of Traditional Dry Sausages from Northeast China. Food Microbiol..

[35] Zhao Y., Wei W., Tang L., Wang D., Wang Y., Wu Z., Zhang W. (2021). Characterization of Aroma and Bacteria Profiles of Sichuan Industrial Paocai by HS-SPME-GC-O-MS and 16S RRNA Amplicon Sequencing. Food Res. Int..

[36] Sabela M.I., Mpanza T., Kanchi S., Sharma D., Bisetty K. (2016). Electrochemical Sensing Platform Amplified with a Nanobiocomposite of L-Phenylalanine Ammonia-Lyase Enzyme for the Detection of Capsaicin. Biosens. Bioelectron..

[37] Wen R., Kong B., Yin X., Zhang H., Chen Q. (2022). Characterisation of Flavour Profile of Beef Jerky Inoculated with Different Autochthonous Lactic Acid Bacteria Using Electronic Nose and Gas Chromatography–Ion Mobility Spectrometry. Meat Sci..

[38] Zhang Q., Ma J., Yang Y., Deng J., Zhu K., Yi Y., Tang J., Jiang X., Zhu C., Laghi L. (2023). Effects of S. Cerevisiae Strains on the Sensory Characteristics and Flavor Profile of Kiwi Wine Based on E-Tongue, GC-IMS and 1H-NMR. LWT.

[39] Liu H., Yu Y., Zou B., Yu Y., Yang J., Xu Y., Chen X., Yang F. (2023). Evaluation of Dynamic Changes and Regularity of Volatile Flavor Compounds for Different Green Plum (*Prunus mume* Sieb. et Zucc) Varieties during the Ripening Process by HS-GC–IMS with PLS-DA. Foods.

[40] Wang D., Zhang J., Zhu Z., Lei Y., Huang S., Huang M. (2022). Effect of Ageing Time on the Flavour Compounds in Nanjing Water-Boiled Salted Duck Detected by HS-GC-IMS. LWT.

[41] Box G.E.P., Cox D.R. (1964). An Analysis of Transformations. J. R. Stat. Soc. Ser. B (Methodol.).

