# Supplementary material for: Effect of Capsaicin Stress on Aroma-Producing Properties of Lactobacillus plantarum CL-01 Based on E-Nose and GC–IMS
Source: Molecules. 2023 Dec 23;29(1):107. doi: 10.3390/molecules29010107 (PMC10780002; doi:10.3390/molecules29010107)
Supplement: Supplementary file 1 [file molecules-29-00107-s001.zip › molecules-2764688-supplementary.pdf]

Effect of Capsaicin Stress on Aroma-Producing Properties of *Lactobacillus plantarum* CL-01 Based on E-Nose and GC–IMS

Qian Zhang [1], Junni Tang [1], Jing Deng [2], Zijian Cai [1], Xiaole Jiang [3] and Chenglin Zhu [1,*]

1 College of Food Science and Technology, Southwest Minzu University, Chengdu 610041, China; zhangqian1121@outlook.com (Q.Z.); junneytang@swun.edu.cn (J.T.); caizijian@swun.edu.cn (Z.C.)
2 Cuisine Science Key Laboratory of Sichuan Province, Sichuan Tourism University, Chengdu 610100, China; dj3930590@sina.com
3 College of Chemistry and Environment, Southwest Minzu University, Chengdu 610041, China; jiangxl@swun.edu.cn
* Correspondence: chenglin.zhu@swun.edu.cn; Tel.: +86-028-85928478

Supporting Information

Table S1. Response values (mean ± sd) of the E-nose to *L. plantarum* fermentation for 24 h under different capsaicin concentrations.

Sensors	A	B	C	D
LY2/LG	$2.83\times10^{-2} \pm 2.90\times10^{-2}$	$1.17\times10^{-2} \pm 1.61\times10^{-3}$	$1.27\times10^{-2} \pm 3.72\times10^{-3}$	$1.15\times10^{-2} \pm 2.98\times10^{-3}$
LY2/G	$-2.95\times10^{-2} \pm 2.43\times10^{-2}$	$-1.78\times10^{-2} \pm 2.70\times10^{-3}$	$-1.86\times10^{-2} \pm 4.27\times10^{-3}$	$-1.72\times10^{-2} \pm 3.19\times10^{-3}$
LY2/AA	$-8.76\times10^{-3} \pm 9.30\times10^{-3}$	$-4.03\times10^{-3} \pm 5.11\times10^{-4}$	$-4.57\times10^{-3} \pm 1.30\times10^{-3}$	$-4.10\times10^{-3} \pm 8.97\times10^{-4}$
LY2/Gh	$-1.57\times10^{-2} \pm 1.18\times10^{-2}$	$-1.05\times10^{-2} \pm 1.93\times10^{-3}$	$-1.08\times10^{-2} \pm 2.52\times10^{-3}$	$-9.85\times10^{-3} \pm 1.81\times10^{-3}$
LY2/gCTl	$-1.93\times10^{-2} \pm 1.29\times10^{-2}$	$-1.45\times10^{-2} \pm 2.13\times10^{-3}$	$-1.56\times10^{-2} \pm 3.85\times10^{-3}$	$-1.41\times10^{-2} \pm 2.72\times10^{-3}$
LY2/gCT	$-1.08\times10^{-2} \pm 5.72\times10^{-3}$	$-1.04\times10^{-2} \pm 9.06\times10^{-4}$	$-1.30\times10^{-2} \pm 4.09\times10^{-3}$	$-1.13\times10^{-2} \pm 3.05\times10^{-3}$
T30/1	$9.82\times10^{-2} \pm 3.43\times10^{-3}$	$9.41\times10^{-2} \pm 5.46\times10^{-4}$	$9.40\times10^{-2} \pm 3.40\times10^{-4}$	$9.44\times10^{-2} \pm 6.19\times10^{-4}$
P10/1	$9.30\times10^{-2} \pm 1.91\times10^{-3}$	$9.27\times10^{-2} \pm 2.21\times10^{-4}$	$9.25\times10^{-2} \pm 1.54\times10^{-4}$	$9.28\times10^{-2} \pm 4.13\times10^{-4}$
P10/2	$8.56\times10^{-2} \pm 1.63\times10^{-3}$	$8.47\times10^{-2} \pm 1.37\times10^{-3}$	$8.59\times10^{-2} \pm 1.52\times10^{-3}$	$8.56\times10^{-2} \pm 7.24\times10^{-4}$
P40/1	$8.67\times10^{-2} \pm 1.68\times10^{-3}$	$8.80\times10^{-2} \pm 7.78\times10^{-4}$	$8.84\times10^{-2} \pm 1.31\times10^{-3}$	$8.79\times10^{-2} \pm 1.11\times10^{-3}$
T70/2	$9.60\times10^{-2} \pm 3.97\times10^{-3}$	$9.17\times10^{-2} \pm 3.82\times10^{-4}$	$9.19\times10^{-2} \pm 1.54\times10^{-5}$	$9.19\times10^{-2} \pm 8.26\times10^{-5}$
PA/2	$1.01\times10^{-1} \pm 2.84\times10^{-3}$	$9.78\times10^{-2} \pm 7.86\times10^{-4}$	$9.74\times10^{-2} \pm 1.18\times10^{-3}$	$9.83\times10^{-2} \pm 1.35\times10^{-3}$
P30/1	$1.03\times10^{-1} \pm 2.94\times10^{-3}$	$9.86\times10^{-2} \pm 1.60\times10^{-3}$	$9.80\times10^{-2} \pm 2.05\times10^{-3}$	$9.92\times10^{-2} \pm 2.45\times10^{-3}$
P40/2	$9.03\times10^{-2} \pm 5.21\times10^{-3}$	$8.62\times10^{-2} \pm 1.40\times10^{-4}$	$8.68\times10^{-2} \pm 1.05\times10^{-3}$	$8.61\times10^{-2} \pm 7.08\times10^{-4}$
P30/2	$1.01\times10^{-1} \pm 4.03\times10^{-3}$	$9.57\times10^{-2} \pm 1.00\times10^{-3}$	$9.51\times10^{-2} \pm 1.17\times10^{-3}$	$9.56\times10^{-2} \pm 1.42\times10^{-3}$
T40/2	$6.93\times10^{-2} \pm 5.10\times10^{-3}$	$7.00\times10^{-2} \pm 1.96\times10^{-3}$	$7.12\times10^{-2} \pm 3.56\times10^{-3}$	$6.94\times10^{-2} \pm 2.90\times10^{-3}$
T40/1	$7.05\times10^{-2} \pm 3.18\times10^{-3}$	$7.70\times10^{-2} \pm 3.16\times10^{-3}$	$7.75\times10^{-2} \pm 3.59\times10^{-3}$	$7.56\times10^{-2} \pm 3.48\times10^{-3}$
TA/2	$6.07\times10^{-2} \pm 3.44\times10^{-3}$	$6.92\times10^{-2} \pm 4.59\times10^{-3}$	$7.12\times10^{-2} \pm 6.16\times10^{-3}$	$6.82\times10^{-2} \pm 6.10\times10^{-3}$

Table S2. Response values (mean ± sd) of the E-nose to *L. plantarum* along fermentation under capsaicin stress

Sensors	BT00	BT06	BT12	BT18	BT24
LY2/LG	$6.64\times10^{-3} \pm 5.45\times10^{-4}$	$1.14\times10^{-2} \pm 6.91\times10^{-3}$	$1.85\times10^{-2} \pm 1.58\times10^{-2}$	$1.19\times10^{-2} \pm 3.88\times10^{-4}$	$2.14\times10^{-2} \pm 8.89\times10^{-4}$
LY2/G	$-1.18\times10^{-2} \pm 3.86\times10^{-4}$	$-2.05\times10^{-2} \pm 1.18\times10^{-2}$	$-2.88\times10^{-2} \pm 2.22\times10^{-2}$	$-1.67\times10^{-2} \pm 3.98\times10^{-4}$	$-3.02\times10^{-2} \pm 6.01\times10^{-4}$
LY2/AA	$-2.35\times10^{-3} \pm 2.06\times10^{-4}$	$-4.45\times10^{-3} \pm 2.57\times10^{-3}$	$-7.36\times10^{-3} \pm 5.89\times10^{-3}$	$-4.14\times10^{-3} \pm 7.01\times10^{-4}$	$-5.04\times10^{-3} \pm 5.15\times10^{-4}$
LY2/Gh	$-8.53\times10^{-3} \pm 2.62\times10^{-4}$	$-1.51\times10^{-2} \pm 1.04\times10^{-2}$	$-2.15\times10^{-2} \pm 2.00\times10^{-2}$	$-1.25\times10^{-2} \pm 3.73\times10^{-4}$	$-1.76\times10^{-2} \pm 3.40\times10^{-4}$
LY2/gCTl	$-1.22\times10^{-2} \pm 6.50\times10^{-4}$	$-1.76\times10^{-2} \pm 8.55\times10^{-3}$	$-2.42\times10^{-2} \pm 1.75\times10^{-2}$	$-1.50\times10^{-2} \pm 2.40\times10^{-3}$	$-2.24\times10^{-2} \pm 2.20\times10^{-4}$
LY2/gCT	$-8.99\times10^{-3} \pm 5.29\times10^{-4}$	$-1.39\times10^{-2} \pm 8.39\times10^{-3}$	$-2.26\times10^{-2} \pm 2.09\times10^{-2}$	$-1.17\times10^{-2} \pm 1.03\times10^{-3}$	$-1.24\times10^{-2} \pm 1.32\times10^{-3}$
T30/1	$9.58\times10^{-2} \pm 2.07\times10^{-3}$	$1.45\times10^{-1} \pm 8.59\times10^{-2}$	$1.80\times10^{-1} \pm 1.49\times10^{-1}$	$9.39\times10^{-2} \pm 1.82\times10^{-4}$	$9.40\times10^{-2} \pm 4.14\times10^{-4}$
P10/1	$9.28\times10^{-2} \pm 2.41\times10^{-3}$	$1.09\times10^{-1} \pm 3.12\times10^{-2}$	$1.17\times10^{-1} \pm 4.24\times10^{-2}$	$9.25\times10^{-2} \pm 3.75\times10^{-4}$	$9.97\times10^{-2} \pm 1.13\times10^{-3}$
P10/2	$8.67\times10^{-2} \pm 2.64\times10^{-3}$	$9.85\times10^{-2} \pm 2.47\times10^{-2}$	$8.34\times10^{-2} \pm 2.84\times10^{-3}$	$8.57\times10^{-2} \pm 3.45\times10^{-4}$	$8.46\times10^{-2} \pm 1.28\times10^{-3}$
P40/1	$8.64\times10^{-2} \pm 3.73\times10^{-3}$	$1.03\times10^{-1} \pm 2.67\times10^{-2}$	$1.18\times10^{-1} \pm 5.13\times10^{-2}$	$8.84\times10^{-2} \pm 2.29\times10^{-4}$	$9.91\times10^{-2} \pm 1.47\times10^{-3}$
T70/2	$8.64\times10^{-2} \pm 4.86\times10^{-3}$	$9.44\times10^{-2} \pm 3.92\times10^{-3}$	$9.64\times10^{-2} \pm 8.47\times10^{-3}$	$9.18\times10^{-2} \pm 3.83\times10^{-4}$	$9.50\times10^{-2} \pm 6.11\times10^{-3}$
PA/2	$9.88\times10^{-2} \pm 6.92\times10^{-4}$	$1.11\times10^{-1} \pm 2.00\times10^{-2}$	$1.09\times10^{-1} \pm 1.96\times10^{-2}$	$9.74\times10^{-2} \pm 3.16\times10^{-4}$	$9.77\times10^{-2} \pm 7.16\times10^{-4}$
P30/1	$9.74\times10^{-2} \pm 3.51\times10^{-3}$	$7.02\times10^{-2} \pm 5.32\times10^{-2}$	$6.67\times10^{-2} \pm 5.55\times10^{-2}$	$9.77\times10^{-2} \pm 5.71\times10^{-4}$	$9.85\times10^{-2} \pm 1.50\times10^{-3}$
P40/2	$8.70\times10^{-2} \pm 1.14\times10^{-3}$	$6.14\times10^{-2} \pm 4.29\times10^{-2}$	$5.94\times10^{-2} \pm 4.54\times10^{-2}$	$8.60\times10^{-2} \pm 4.15\times10^{-4}$	$8.61\times10^{-2} \pm 6.69\times10^{-5}$
P30/2	$9.52\times10^{-2} \pm 2.05\times10^{-3}$	$8.02\times10^{-2} \pm 2.93\times10^{-2}$	$7.67\times10^{-2} \pm 3.28\times10^{-2}$	$9.51\times10^{-2} \pm 3.59\times10^{-4}$	$9.56\times10^{-2} \pm 8.96\times10^{-4}$
T40/2	$6.99\times10^{-2} \pm 2.77\times10^{-3}$	$5.38\times10^{-2} \pm 2.48\times10^{-2}$	$5.44\times10^{-2} \pm 2.61\times10^{-2}$	$7.05\times10^{-2} \pm 1.22\times10^{-3}$	$6.99\times10^{-2} \pm 2.05\times10^{-3}$
T40/1	$7.58\times10^{-2} \pm 3.79\times10^{-3}$	$6.84\times10^{-2} \pm 1.01\times10^{-2}$	$5.91\times10^{-2} \pm 3.09\times10^{-2}$	$7.78\times10^{-2} \pm 1.42\times10^{-3}$	$7.69\times10^{-2} \pm 3.28\times10^{-3}$
TA/2	$6.49\times10^{-2} \pm 1.67\times10^{-3}$	$6.59\times10^{-2} \pm 4.59\times10^{-3}$	$6.52\times10^{-2} \pm 6.89\times10^{-3}$	$7.13\times10^{-2} \pm 1.33\times10^{-3}$	$6.92\times10^{-2} \pm 4.67\times10^{-3}$
